# Maternity care during COVID-19: a qualitative evidence synthesis of women’s and maternity care providers’ views and experiences

**DOI:** 10.1186/s12884-022-04724-w

**Published:** 2022-05-26

**Authors:** Sarah Jane Flaherty, Hannah Delaney, Karen Matvienko-Sikar, Valerie Smith

**Affiliations:** 1 School of Public Health, University College Cork, Cork, Ireland; 2 School of Nursing and Midwifery, University of Dublin Trinity College Dublin, Dublin, Ireland; 3 Health Research Board-Trials Methodology Research Network (HRB-TMRN), National University of Ireland, Galway, Ireland

**Keywords:** Maternity care, Women's experiences, Maternity care providers, COVID-19, Corona virus, Qualitative evidence synthesis, Systematic review

## Abstract

**Background:**

As COVID-19 continued to impact society and health, maternity care, as with many other healthcare sectors across the globe, experienced tumultuous changes. These changes have the potential to considerably impact on the experience of maternity care. To gain insight and understanding of the experience of maternity care during COVID-19, from the perspectives of women and maternity care providers, we undertook a qualitative evidence synthesis (QES).

**Methods:**

The population of interest for the QES were pregnant and postpartum women, and maternity care providers, who provided qualitative data on their experiences of maternity care during COVID-19. The electronic databases of MEDLINE, CINAHL, EMBASE, PsycINFO and the Cochrane COVID study register were systematically searched from 01 Jan 2020 to 13 June 2021. The methodological quality of the included studies was appraised using a modified version of the quality assessment tool, based on 12-criteria, designed by the Evidence for Policy and Practice Information coordinating Centre (EPPI-Centre). Data were extracted by two reviewers independently and synthesised using the Thomas and Harden framework. Confidence in the findings was assessed using the Grading of Recommendations Assessment, Development and Evaluation-Confidence in the Evidence from Reviews of Qualitative research (GRADE-CERQual).

**Results:**

Fifty records relating to 48 studies, involving 9,348 women and 2,538 maternity care providers, were included in the QES. The methodological quality of the studies varied from four studies meeting all 12 quality criteria to two studies meeting one quality criterion only. The synthesis revealed eight prominent themes. Five of these reflected women’s experiences: 1) Altered maternity care (women), 2) COVID-related restrictions, 3) Infection prevention and risk, 4) ‘the lived reality’ – navigating support systems, and 5) Interactions with maternity services. Three themes reflected maternity care providers’ experiences: 6) Altered maternity care (providers), 7) Professional and personal impact, and 8) Broader structural impact. Confidence in the findings was high or moderate.

**Conclusion:**

Although some positive experiences were identified, overall, this QES reveals that maternity care during COVID-19 was negatively experienced by both women and maternity care providers. The pandemic and associated changes evoked an array of emotive states for both populations, many of which have the potential to impact on future health and wellbeing. Resource and care planning to mitigate medium- and longer-term adverse sequelae are required.

**PROSPERO registration:**

CRD42021232684.

**Supplementary Information:**

The online version contains supplementary material available at 10.1186/s12884-022-04724-w.

## Background

Over two years from being declared a global pandemic, COVID-19 continues to impact society and health. Maternity care, as with many other healthcare sectors across the globe, has experienced tumultuous change. Unlike many other healthcare sectors, however, individuals accessing maternity care, for the most part, are healthy women and their families, with unique healthcare needs that can differ to those who have pathological ill-health. Additionally, in providing maternity care, the health and wellbeing direct needs of two individuals, that is the women and her baby, rather than one individual, must be considered. Changes to healthcare provision, in this sense, can impact the care recipients differently, depending on the health sector concerned. Some of the changes to healthcare as a result of COVID-19 involved a move towards telehealth and remote antenatal and postnatal appointments, redeployment of midwives across the sector, for example, for screening and vaccination, and reduced or altered postnatal support [1–4]. In addition, for many women who gave birth during COVID-19, the majority will have done so in a system that prohibited birth partner attendance at antenatal and postnatal visits. Birth partner presence during labour was also restricted in many places to attending during active labour only or not attending at all, thus reducing birth partners to an ‘unnatural state of a spectator’ [5] p.5].

Although pregnant women are no more likely to contract COVID-19 than other population groups, the risk for pregnancy complications in women who are COVID-19 positive appears heightened. For example, studies have reported increased risks for preterm birth, caesarean birth, and, in rare cases, maternal death [6, 7]. Other common complications reported include intrauterine fetal distress and premature rupture of membranes, shortness of breath and gastrointestinal symptoms [6]; clinical manifestations which may impact and alter women’s care trajectories during their pregnancy, labour and birth, and in the postpartum period. Women’s perinatal emotional wellbeing has also been considerably affected. Pre-pandemic rates of perinatal depression globally were reported at 11.9% [8]. Recent pooled prevalence, based on a rapid review of 46 studies, has cited rates of perinatal depression and anxiety during COVID-19 of 25.6% and 30.5%, respectively, more than double pre-COVID levels [9]. Moreover, anxiety and depression in new mothers who gave birth during COVID-19 was reported as high as 61.9% [10], with rates of clinically relevant depression, up to 12 weeks postpartum, of 43% [11]. The seriousness of the altered systems of care, alongside increased rates of psychological distress was highlighted in a recent Mothers and Babies: Reducing Risk through Audits and Confidential Enquiries across the UK (MBRRACE-UK) report which documented two instances where women died by suicide, as referrals to perinatal mental health services were denied or delayed because of COVID-19 related restrictions [12]. Aside from the immediate impacts, perinatal mental ill-health can continue into the early parenthood years, with potential reverberations for optimal maternal-child bonding, parenting confidence, overall emotional wellbeing, and quality of life.

Midwives, obstetricians, and other allied maternity care providers have also experienced significant challenges during the COVID-19 pandemic. Adapting, in many cases overnight, to an altered system of care, maternity care providers experienced fear of the unknown, unpreparedness and fear of contracting COVID-19 [13–15]. Access to essential equipment, such as personal protective equipment (PPE), especially in the early days of the pandemic, also presented as concerning and stress-inducing issues for maternity care providers [16, 17]. Coordinating home life with work life, especially during periods of national lockdown, coupled with a fear of infecting family members because of exposure to the virus at work, will have also affected the wellbeing of those providing maternity care.

Although the global vaccination programme has offered optimism and a sense of anticipation that approaches to tackling the coronavirus are moving in a positive direction, new variants of COVID-19 continue to emerge. As a result, health care advisors and the community at large remain on heightened alert, and global healthcare continues to be affected, including that of maternity care. Understanding the experiences of those directly involved in receiving and providing maternity care during COVID-19 is critically important for optimising quality care as the pandemic continues and beyond. As qualitative studies exploring stakeholder experiences of maternity care, from across the globe, are being made available, bringing the findings together from these studies through evidence synthesis will help establish a greater understanding of the emerging issues from the perspectives of those directly involved. For this reason, we conducted a qualitative evidence synthesis (QES) of pregnant and postpartum women’s and maternity care providers’ views and experiences of maternity care during COVID-19. By collating the existing evidence, the findings from this QES will uncover new information and create an awareness of the impact of COVID-19 from the perspectives of those directly affected, which may guide resource and care needs, including mental health care needs, now and into the future.

## Methods

This QES is registered with PROSPERO (CRD42021232684) and the protocol is published and openly available [18]. The protocol adheres to the Enhancing transparency in reporting the synthesis of qualitative research (ENTREQ) guideline [19] (completed checklist available at: https://osf.io/bzt38/).

### Inclusion criteria

The inclusion criteria are defined in our protocol [18]. In brief, using the SPIDER (Sample, Phenomenon of Interest, Design, Evaluation, and Research type) acronym [20], the **S**ample was primiparous and multiparous women who were pregnant or up to six months postpartum at the time of study, and maternity care providers (midwives, obstetric nurses, obstetricians, doctors, and allied maternity care professionals) who were directly involved in maternity care provision during the COVID-19 pandemic. The **P**henomenon of **I**nterest was women’s and maternity care providers experiences of maternity care during COVID-19. For this QES maternity care is broadly defined as the care provided, inclusive of health and wellbeing monitoring and assessments and the provision of perinatal health education and information to women, babies, and their families during pregnancy, labour and childbirth and in the postpartum period, up to six weeks following childbirth. Care settings may be the hospital, community, or home birth settings. Study **D**esigns were published and unpublished qualitative studies and studies of mixed methods design where the qualitative data could be extracted separately. Survey designs with free-text response options were also considered for inclusion if the available qualitative data were of sufficient depth and had been analysed formally using a structured approach (e.g., thematic analysis, content analysis, etc.). The **E**valuation of outcomes was centred on the narrative views, experiences, and perspectives of pregnant and postpartum women and maternity care providers. The **R**esearch type included primary research studies, in the English language, available from 01 January 2020 to the date of our search. Study abstracts were also considered if they reported sufficient data to contribute to the synthesis in a meaningful way.

### Search strategy

To identify eligible studies, the electronic databases of MEDLINE, CINAHL, EMBASE, PsycINFO and the Cochrane COVID study register (https://covid-19.cochrane.org) were systematically searched from 01 Jan 2020 to 22 Feb 2021. Given the pandemic context and the rapidity with which new studies were becoming available, we updated our searches on 13 June 2021 prior to commencing data synthesis. To avoid potential misrepresentations arising from language and contextual nuances in translating text, non-English full-text publications were excluded from the QES, however we included all languages in our search strategy. This allowed us to identify the extent of potentially eligible non-English publications and whether this presented as a source of possible language bias. The search terms, and their combinations, which were guided by our SPIDER inclusion criteria and adapted as relevant for database specific subject terms, were detailed in our protocol [18] and independently peer reviewed prior to implementation. These search terms were:

- **S:** mother OR woman OR women OR midwives OR midwife* OR nurs* OR clinician OR physician OR doctor OR obstetric* OR professional AND
- **PI:** (maternity ADJ care) OR healthcare OR ‘health-care’ OR matern* OR birth* OR childbirth OR prenan* OR labour OR labor OR antenatal OR antepartum OR postnatal OR postpartum OR post-partum OR puerperium AND coronavirus* OR corona virus* OR COVID-19 OR COVID OR covid OR Covid2019 OR SARS-CoV* OR SARSCov* OR new CoV* OR novel CoV* AND
- **E** and **R:** experiences OR experience OR view* OR perceptions OR perception OR voices OR narratives OR qualitative OR (mixed ADJ method) OR ‘grounded theory’ OR phenomenology OR ‘action research’.

To further enhance sensitivity, we extended our search to include searches of the reference lists of included studies, the grey literature websites of http://www.opengrey.eu/ and https://greylit.org and the proceedings of the international Normal Labour and Birth Research Conference (Dec 2020) and the Maternity Expo Conference: Maternity Services after COVID-19 (Sept 2020).

### Study selection

Once all searches were complete and the citations were exported to EndNote reference manager, duplicate citations were removed. The remaining records were uploaded to Covidence, a software package designed to assist with preparing systematic reviews. Three members of the review team (SJF, KMS and VS) screened the records on title and abstract, with each record independently screened by at least two reviewers. Records forwarded for full text review were independently screened by two reviewers (SJH and VS). Disagreements at each stage of the selection process were resolved through discussion and consensus.

### Quality assessment

An adapted version of a quality appraisal tool developed by the Evidence for Policy and Practice Information and Co-ordinating (EPPI) Centre for use in a systematic review of healthy eating in children [21] and used in previous QES by review authors [22, 23], was used to assess the methodological quality of the included studies. Using the tool, each included study was assessed independently by pairs of reviewers (SJF and VS; KMS and HD) on the extent to which the study met the tool’s 12 quality appraisal criteria. Minimum standards for a Yes, No or Partially met judgement were agreed in advance (Supplementary File 1). The 12 assessment criteria spanned three domains: i) the quality of the study reporting, ii) the reliability and validity of data collection and analysis, and iii) the quality of the study methods. A decision to include all studies following quality assessments was agreed because qualitative data providing perspectives on views and experiences, irrespective of methodological quality “…could have led to important new angles of consideration” [24] p.1718].

### Data extraction and synthesis

Data were extracted from the included studies using a pre-designed data extraction form (see https://osf.io/bzt38/ for the template form). The form was initially piloted on two studies and would have been refined, if necessary, but this was not required. Relevant data were extracted from each study independently in pairs (SJF and VS; KMS and HD) and cross-checked for accuracy. The following information was extracted as available: study reference (including publication type and year published), study aim, description of the participants and the study setting, dates when the study was conducted, data collection and analysis methods, funding details, and all findings related to pregnant and postpartum women’s and maternity care providers’ views of maternity care during COVID-19. For studies that reported on both women’s and maternity care providers’ views and experiences, the data were extracted and tagged to the relevant population category for synthesis.

Thomas and Harden’s thematic synthesis framework, which involves line by line coding of extracted text, developing descriptive themes and generating analytical themes, was used to guide the synthesis of the findings data [25]. This synthesis method was chosen over other methods (e.g., framework synthesis, meta-ethnography), as it provides a process that can be used for synthesising the findings from most, if not all qualitative enquiries [25], and allows for the inductive identification and development of themes that reflect the included studies data, overall. To enhance rigour, two members of the review team (SJF and VS) independently coded data from three included studies, initially, and met to compare the codes for consistency and congruity. Following this, the extracted data were categorised into women’s data and maternity care providers’ data. These category data were coded separately by two reviewers (VS for women’s data and SJF for providers’ data), using the comment function in Microsoft Word to add codes to the text, and the descriptive themes were developed. A meeting involving all four review authors was then held where the descriptive themes and associated codes were reviewed, refined (if required) and agreed based on discussion, reflection, and iteration. A similar process was used in determining the analytical themes; that is, one reviewer (VS) developed the analytical themes relating to women’s views and experiences, one reviewer (SJF) developed the analytical themes relating to maternity care providers’ views and experiences, and all members of the review team met to discuss and agree the final analytical themes.

### Assessment of confidence in the review findings

#### GRADE-CERQual

The Grading of Recommendations Assessment, Development and Evaluation-Confidence in the Evidence from Reviews of Qualitative research (GRADE-CERQual) was used to assess the confidence in the QES findings [26–31]. Using GRADE-CERQual, each discrete finding identified in the synthesis was assessed on i) the methodological limitations of the studies contributing to the finding, ii) the coherence of the finding, iii) the adequacy of data contributing to the finding and iv) the relevance of the contributory studies to the review question. We set an initial assumption of ‘High confidence’ in all findings and downgraded accordingly if judged appropriate based on the criteria described in our QES protocol [18]. The assessment of each finding was carried out independently by at least two reviewers with final judgements based on discussion and consensus. An overall judgement of High, Moderate, Low or Very Low confidence in each finding was then agreed [26].

## Results

### Search and selection

**Fig. 1:**
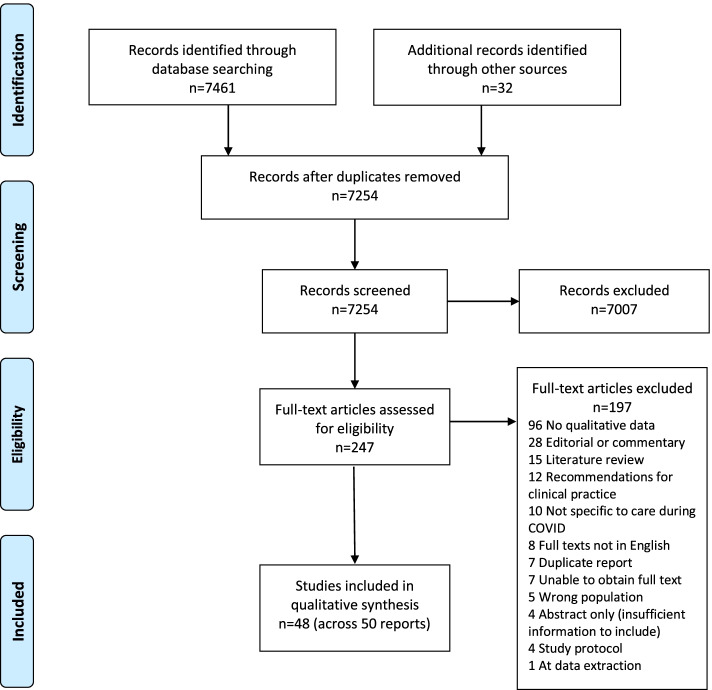
Search and selection flow diagram

The database searches yielded 7461 records, with a further 32 records identified from searching additional sources. Of these 7493 records, 239 were duplicates and removed. The resulting 7254 records were screened on title and abstract against the QES inclusion criteria; 7007 of these were clearly ineligible and excluded. Full texts of the remaining 247 were retrieved and assessed for eligibility, of which 196 were excluded. During data extraction, one further study was subsequently excluded as it became clear that the sample were not maternity care providers and the data related to women’s views were not specific to COVID-19 [32]. Details of these 197 excluded records with reasons is available at https://osf.io/bzt38/. This screening process resulted in the inclusion of 50 records reporting on 48 studies, of which 32 were included based on our initial search (Feb 2021) and 16 included following our updated search [2, 15, 17, 33–79]. For three records arising from one study, each of the records reported on a discrete population, that is, doctors [73], midwives [59] and women [56], with nuanced methods for recruitment and data collection as applicable to each population. For quality assessment, data extraction and synthesis purposes, we thus considered these records as single ‘studies’ contributing to the QES. Twenty-seven of the included 50 records provided data from pregnant and postpartum women, 17 provided data from maternity care providers and six provided data from both populations (Table 1). The screening and selection process, including results, is presented in Fig. 1 using the PRISMA flowchart [80].

**Table 1:** Descriptive characteristics of included studies

Ref No	Aim	Country	Description of participants	Number
**Studies contributing data on women’s views and experiences**				
33	Personal narrative of experiences during COVID-19	UK	Primiparous woman with gestational diabetes accessing public maternity care	*N* = 1
34	To study the perspectives of pregnant women in relation to the impact of the COVID-19 pandemic on their pregnancy experience	Australia	Pregnant women any gestation booked and receiving antenatal care at the hospital	*N* = 15
35	To investigate the pregnancy experiences of women during the COVID-19 pandemic	Turkey	Pregnant women admitted to the study site, > 20 years old, communicating in Turkish and not COVID-19 positive (*n* = 14)	*N* = 14
36	To assess stressors, coping behaviors, and resources needed in relation to the COVID-19 pandemic in a sample of perinatal women in the United States	USA	Pregnant or postpartum women, ≥ 18 years of age, English-speaking, and gave birth between Jan and May 2020	*N* = 42*
37	To describe the impact of the COVID-19 pandemic on birth experiences centering the perspective of the birthing person	USA	Women ≥ 18 years of age who gave birth after 01 Mar 2020 in the US using the Ovia Parenting app	N = 202^&^
38	Personal narrative of experiences during COVID-19	UK	Postpartum woman of twins	*N* = 1
39	To better understand the care that women are receiving and/or seeking in response to COVID-19 in order to inform Government and other key stakeholders	Australia	All women who were pregnant in Australia	*N* = 2750^+^
40	To gain an understanding of women’s experiences of visiting restrictions imposed due to COVID-19 and to provide information to inform policy development in relation to visiting	Ireland	Women during the antenatal period in a large urban maternity unit	*N* = 303^&^
41	To explore the experience of expectant parents who accessed hypnobirthing online classes during the COVID-19 pandemic	UK	Pregnant or postpartum women	*N* = 25
42	To better understand mental health and well-being, as well as sources of resilience, for women in the perinatal period during the COVID-19 pandemic	USA	Women > 18 years, English-speaking, currently living in Colorado, and being pregnant or within the first 6-months postpartum	*N* = 31
43	To examine the impact of COVID on patients' access and utilization of prenatal genetic screens and diagnostic tests at the onset of the COVID‐19 pandemic	USA	Pregnant women in first and second trimesters	*N* = 40
44	To explore childbearing experiences of COVID-19 positive mothers who gave birth in a Northern Italy maternity hospital	Italy	All women who tested positive for COVID-19 at the research site during the months of Mar and Apr 2020	*N* = 22
45	To identify challenges with healthcare interactions experienced by postpartum patients during the pandemic	USA	Postpartum women: the median time between birth and the interviews was 10 weeks	*N* = 40
46	To explore if and how women perceived their prenatal care to have changed due to COVID-19 and the emotional impact of those changes on pregnant women	USA	Pregnant women able to complete an online survey in English, regardless of gestational age of the pregnancy, location of residence or utilization of services	*N* = 2519
47	To explore pregnant women’s perceptions of COVID-19 and their healthcare experiences	UK	Women currently pregnant or postpartum since the COVID-19 pandemic commenced	*N* = 1451
48	To capture peripartum women's lived experiences during the COVID-19 pandemic	India	Pregnant women > 30th week of gestation to 1-month postpartum who could speak English or Hindi language	*N* = 25
49	To assess pregnant women’s satisfaction with antenatal care and social support and to examine stress-reduction strategies women used during the pandemic	International	Pregnant women aged 18 years or older irrespective of gestational age, nationality, or geographical location	*N* = 558^&^
50	To explore the lived experiences of pregnant women during the COVID-19 pandemic to better understand their experience of pregnancy so that better support could be provided	Iran	Pregnant women who were registered in public health centers affiliated with Sabzevar University of Medical Sciences	*N* = 19
2	To gain insight and understanding of women’s experiences of maternity care during the first national lock-down phase of COVID-19 in one hospital setting	Ireland	Women ≥ 18 years of age, able to read and speak English, and had experienced pregnancy, childbirth (between 37 and 42 weeks of pregnancy) and postnatal care at the study site during the first national lock-down period	*N* = 19
51	To investigate how COVID-19 and associated restrictions influence mood and parenting confidence of expectant parents and those in early parenthood	UK	Women currently pregnant or postpartum	*N* = 564^$^
53	To gain insights into the attitudes and experiences of expectant and recent parents during COVID-19	UK	Women who were Baby Buddy App users, irrespective of their gestational stage and baby age < 24 weeks	*N* = 32^&^
53	To understand the experiences of pregnant women during the COVID-19 pandemic	Turkey	Pregnant women > 18 years who speak Turkish, not diagnosed with COVID-19 and are residents of Turkey	*N* = 15
54	To explore perceptions of social support among breastfeeding mothers during the COVID-19 pandemic	Not stated	Postpartum women currently breastfeeding	*N* = 29
55	To better understand the ways in which new families experience pregnancy and lactation during the COVID-19 pandemic	USA	Healthy first-time mothers with a prenatal intention to breastfeed	N = 3
56	To describe childbearing women’s experiences of becoming a mother during the first wave of the COVID-19 pandemic	Australia	Women of different ethnicities living in varied geographical locations across Australia, and seeking care from a wide variety of models of care	*N* = 27^&^
57	To describe lived experience in COVID -19 lockdown period from the perspective of pregnant women	India	Pregnant women in any trimester	*N* = 4
58	Personal narrative of experiences during COVID-19	UK	One multiparous woman	*N* = 1
**Studies contributing data on maternity care professionals’ views and experiences**				
59	To explore and describe midwives’ experiences of providing maternity care during the COVID-19 pandemic	Australia	Registered midwives who had provided maternity care since March 2020	*N* = 16^&^
60	Personal narrative of lived experience during COVID-19	Not stated	Resident on a labour and delivery ward	*N* = 1
61	Real time experiences of providing care, services and programming to directly address the needs of pregnant and parenting New Yorkers during COVID-19	USA	Not explicitly described (maternity care providers)	*N* = 9
15	Narrative description of lived experiences	USA	Obstetricians-gynecologists	*N* = 2
62	To analyze how the nurse-midwives of maternity wards have reorganized care in the context of labor and birth amidst the COVID-19 pandemic	Brazil	Nurse-midwife preceptors and collaborators of maternity wards that were fields of practice of the Enhancement Course for Nurse-Midwives	*N* = 9
63	To assess how obstetrics and gynecology NCHDs viewed and were affected by measures taken in response to Covid-19 pandemic	Ireland	Non-consultant hospital doctors in maternity units	*N* = 74
64	Maternal and newborn health professionals experience of providing care to pregnant and postpartum women and their newborns using telemedicine during the COVID-19 pandemic	International	Midwives, nurses, obstetricians, gynecologists, neonatologists, and other maternity health professionals working in urban and rural settings	*N* = 1060
65	To investigate the experiences and attitudes of midwives who have provided pregnancy and childbirth care to women with a confirmed or suspected COVID-19 infection	Spain	Midwives who provided pregnancy and childbirth care to women with a confirmed or suspected COVID-19 infection (average experience of 8 years working as a midwife)	*N* = 14
17	To explore the experience of private practicing midwives in relation to the response to planning for the COVID-19 pandemic	Australia	Midwives currently providing any type of private midwifery services for antenatal, labour and birth and/or postnatal services	*N* = 103
66	To describe nurses’ experiences of caring for perinatal women and newborns during the pandemic	South Korea	Registered Nurses working in hospitals that had confirmed or suspected COVID-19 cases	*N* = 24
67	To determine to what degree prenatal care was able to be transitioned to telehealth during COVID-19 and describe providers’ experience with this transition	USA	All providers who conducted telehealth visits during the implementation period	*N* = 11^&^
68	To document the experiences of Black birth workers supporting pregnant and birthing people and new mamas during the first six months of COVID-19	USA	Black maternity care providers	*N* = 38
69	To understand how COVID-19 has impacted childbirth	Puerto Rico	Puerto Rican women working in the fields of reproductive health	*N* = 11
70	To evaluate the provision of obstetrics and gynecology services during the acute phase of COVID-19	UK	Junior doctors in obstetrics and gynecology across all training units in the NHS	*N* = 148
71	To determine changes to breastfeeding support services during the coronavirus-2019 pandemic according to trained lactation providers	USA	MCPs currently offering breastfeeding services to pregnant/postpartum women, had formal training to provide support, and were over the age of 18	*N* = 39
72	To prospectively document experiences of frontline maternal and newborn healthcare providers	International	Any health professionals directly providing maternal or newborn care, from various countries, contexts, services and facilities at the early stage of the COVID-19 pandemic	*N* = 714
73	To explore and describe doctors’ experiences of providing maternity care during the COVID-19 pandemic	Australia	Medical practitioners who provided care across any part of the antenatal, labour and postnatal continuum since March 2020	*N* = 8^&^
**Studies contributing data on both women’s and professionals’ views and experiences**				
74	To explore how experiences of pregnancy and birth were impacted by the COVID-19 pandemic, both from the patients’ and nurses’ perspectives to understand the multifaceted and intersectional impacts from these adaptations	USA	Women: people who were pregnant or had given birth since Mar 2020, restricted to those living in Washington State MCPs: Registered nurses currently working in a perinatal setting since March 2020 from across the US	*N* = 15 (women) *N* = 14 (MCPs)
75	To describe the hospitalization and early postpartum psychological experience for asymptomatic obstetric patients tested for SARS-CoV-2 and to report the impact of on labor and delivery health care workers’ job satisfaction and workplace anxiety	USA	Women: All women presenting for obstetric care at the two hospitals during the recruitment period MCPs: on obstetric units in the two hospitals	*N* = 318 (women) *N* = 158 (MCPs)
76	To describe the short-term impacts of the COVID-19 pandemic and hints at its potential long-term effects	Italy	Women: who had given birth during or immediately after lockdown (Mar-May 2020) MCPs: midwives active in the city of Bologna	*N* = 49 (women) *N* = 18 (MCPs)
77	To explore COVID-19 related factors influencing ANC service uptake	Ethiopia	Pregnant women: who did not attend all recommended ANC visits, third trimester and above, able to speak the local language, age group 18 to 45 MCPs: working in facilities in selected districts	*N* = 44 (women) *N* = 9 (HCPs)
78	To describe how indigent mothers have responded to and coped with the dramatic changes that have occurred in birth practices as a result of this pandemic	Keyna	Women: mothers who were either expectant or gave birth during the COVID-19 pandemic MCPs: matrons (nurse-midwives who serve as department heads) in charge of maternal health services and traditional midwives	*N* = 20 (women) *N* = 5 (MCPs)
79	To evaluate initial adoption and patient and provider care experience with a COVID-19 prenatal care model at a single institution	USA	Pregnant women: All patients at > 20 weeks’ gestation who were receiving prenatal care MCPs: obstetricians, gynecologists, maternal–fetal medicine physicians, family medicine physicians, and certified nurse midwives	*N* = 150^&^ (women) *N* = 53^&^ (MCPs)

^a^ sub-set of pregnant women only as postpartum included women up to 12 months postpartum; ^&^sub-set who provided qualitative data only; ^$^Data from female pregnant participants used; ^+^Ongoing survey; numbers who have completed to date providing data; Abbreviations: *MCPs* Maternity Care Professionals

### Description of included studies

The summary descriptive characteristics of the included studies, organised by population groups and alphabetically, are presented in Table 1. The summary methodological characteristics of the studies, similarly organised, are presented in Supplementary File 2. A total of 9,348 women and 2,538 maternity care providers were included in the 50 records and contributed data to the QES. The studies spanned the globe with 16 conducted in the USA, seven in the UK, six in Australia, three in Ireland, two in Italy, Turkey and India, and one in each of Iran, Spain, Brazil, Kenya, Ethiopia, South Korea and Puerto Rico. A further three studies were international studies, involving up to 81 countries, and for two, the country of origin is unknown (Table 1). The studies were conducted between December 2019 and December 2020, with the majority (*n* = 35) carried out between March and August 2020. The data collection methods were semi-structured interviews (21 studies) mainly conducted remotely via telephone, Zoom or other such platforms, questionnaires with open-text response options (16 studies), personal narratives of lived experiences (six studies), focus group discussions and individual semi-structured interviews (two studies), unstructured interviews (two studies), and in the remaining three studies, a structured interview, a sharing circle and questionnaires combined with in-depth conversations were respectively used (Supplementary File 2). Data analysis involved thematic analysis in 22 studies, qualitative content analysis in nine studies, personal story telling in six studies, constant comparative method in two studies, Colaizzi’s seven-step content analysis in two studies, framework with thematic analysis in two studies, inductive process consistent with Grounded Theory in one study, the Attride-Sterling Framework in one study, immersion and crystallisation in one study, Giorgi’s four-step phenomenological approach in one study, and for the remaining three studies, although the data were thematically organised, the data analysis method was not explicitly described.

### Quality assessment

Of the 50 records, seven were not subjected to an assessment of methodological quality due to their study design (i.e., case report of lived experiences or study abstract) [33, 38, 54, 58, 60, 61]. The quality of the remaining 43 records ranged from four studies meeting all 12 quality assessment criteria to two studies meeting one quality criterion only. Twenty-five of the studies either fully (Y) or partially (P) met 11 of the 12 criteria because they did not meet criterion L, actively involving the participants in the design and conduct of the study. Table 2 presents the results of the quality assessment process.

**Table 2:** Quality appraisal of included studies (adapted from Thomas [21] et al. 2003)

Quality criteria	
**Quality of the study reporting** A = Aims and objectives clearly reported B = Adequately described the context of the research C = Adequately described the sample and sampling methods D = Adequately described the data collection methods E = Adequately described the data analysis methods	**There was good or some attempt to establish the** F = Reliability of the data collection tools G = Validity of the data collection tools H = Reliability of the data analysis I = Validity of the data analysis
	**Quality of the methods** J = Used the appropriate data collection methods to allow for expression of views K = Used the appropriate methods for ensuring the analysis was grounded in the views L = Actively involved the participants in the design and conduct of the study
**Study**	**Criteria met**
Atmuri 2021 [34]	A, B, C, D, E, F, G, H, I, J, K, L
Aydin 2021 [35]	A, B, C, D, E, F, G, H, I, J, K, L
Barbosa-Leiker 2021 [36]	A, B, C, D, E, F^P^, G^P^, H, I, J^P^, K^P^
Bremen 2020 [37]	A, B, C, D, E, F, G, H, I, J, K, L
Cooper 2021 [39]	A, B^P^, C^P^, D^P^, E^P^, F^P^, G^P^, H^P^, I^P^, J^P^, K^P^
Cullen 2021 [40]	A, B, C^P^, D, E, F^P^, G^P^, H, I, J^P^, K
Einion-Waller 2021 [41]	A^P^, B^P^, C^P^, D^P^, H^P^
Farewell 2020 [42]	A, B, C, D, E, F, G^P^, H, I, J, K
Farrell 2021 [43]	A, B, C, D^P^, E, F, G, H, I, J, K
Fumagalli 2021 [44]	A, B, C, D, E, F, G, H, I, J, K
Gomez-Roas 2021 [45]	A, C^P^, D^P^, E, J, K
Javaid 2021 [46]	A, B, C, D, E, F, G, H^P^, I^P^, J, K^P^
Karavadra 2020 [47]	A, B, C^P^, D, E, F^P^, G^P^, H^P^, I^P^, K^P^
Kumari 2021 [48]	A, B^P^, C, D, E, F, G^P^, H, I, J, K
Meaney 2021 [49]	A, B, C^P^, D, E, F^P^, G^P^, H, I, J^P^, K
Mortazavi 2021 [50]	A, B, C, D, E, F, G^P^, H, I, J^P^, K
Panda 2021 [2]	A, B, C, D, E, F, G, H, I, J, K
Perez 2021 [51]	A, B, C^P^, D, E, F^P^, G^P^, H, I, J^P^, K, L
Rhodes 2020 [52]	A, B, C, D, E, F, G, H, I, J, K, L^P^
Sahin 2021 [53]	A, B, C, D, E, F^P^, G, H, I, J, K
Spatz 2021 [55]	A, B, C^P^, D, E, F, G, H, I, J, K
Sweet 2021 [56]	A, B, C, D, E, F, G^P^, H, I, J, K
Upendra 2020 [57]	A, B^P^, C^P^, D, E, F, G^P^, H, I, J, K
Bradfield 2021 [59]	A, B, C, D, E, F, G, H, I, J, K
Dulfe 2021 [62]	A, B, D^P^, E, F^P^, G^P^, H, I, J^P^, K
Elsayed 2021 [63]	A
Galle 2021 [64]	A, B, C, D, E, F, G^P^, H, I, J, K
Gonzalez-Timoneda 2020 [65]	A, B, C, D, E, F, G, H, I, J, K
Homer 2021 [17]	A, B, C^P^, D, E, H, I, J^P^, K
Kang 2021 [66]	A, B, C, D, E, F, G, H, I, J, K
Madden 2020 [67]	A, B, C^P^, D^P^, E, F^P^, G^P^, H^P^, I, J, K
Oparah 2021 [68]	B, D, E, F, G, H, I, J, K, L^P^
Reyes 2021 [69]	B
Rimmer 2020 [70]	A, B, C^P^, E, H, I^P^, K
Schindler-Ruwisch 2021 [71]	A, B, C^P^, D^P^, E^P^, F^P^, G^P^, H, I, J^P^, K
Semaan 2020 [72]	A^P^, B, C^P^, D^P^, E, F^P^, G^P^, H, I^P^, J, K, L
Szabo 2021 [73]	A, B, C^P^, D^P^, E, F^P^, G^P^, H^P^, I^P^, J^P^, K
Altman 2021 [74]	A, B, C, D, E, F, G, H, I, J, K, L
Bender 2020 [75]	A, B, C^P^, D, E^P^, F^P^, G^P^, H^P^, I^P^, J^P^, K^P^
Bengalia 2021 [76]	A^P^, B, C^P^, D^P^
Hailemariam 2021 [77]	A, B, C^P^, D, E, F, G^P^, H, I, J, K
Ombere 2021 [78]	A^P^, B
Peahl 2021 [79]	A, B, C^P^, D, E^P^, F^P^, G^P^, H^P^, I^P^, J^P^, K^P^, L^P^

*P* Partially met

### Synthesis and findings

The data were synthesised and presented separately by participant category, that is, pregnant and postpartum women and maternity care providers. The totality of the synthesis is represented by eight analytical themes of which five themes and six associated sub-themes represent women’s views and experiences, and three themes and four associated sub-themes represent maternity care providers’ views and experiences. Although there is overlap in some of the themes identified in the participant categories, the themes are presented separately so that the reader can interpret and consider the respective findings explicitly in the context of the participants from which these themes emerged. Table 3 and Table 4 respectively present these themes and the audit trail from the codes (condensed for illustrative purposive) to the descriptive themes and finally to the analytical themes. Codes denoted in bold represent ‘new’ codes from studies that were identified during our updated search. These codes were very few (*n* = 5 for women’s data and *n* = 3 for maternity care providers data), providing reassurance that the inclusion of additional studies is unlikely to alter the overall findings. Illustrative participant quotes from the included studies are presented to support the synthesised findings.

**Table 3:** Theme development for women’s views and experiences

Codes (reduced for illustrative purposes)	Descriptive themes	Analytical sub-themes	Analytical theme
Virtual care worked well; Virtual care problematic; Virtual care	Providing care virtually	*Telehealth*	**Theme 1 Altered maternity care**
New model of care good; **Rushed care;** Changed maternity care; Faster appointments; Cancelled appointments; Varied care; Continuity of care; Increased medicalisation; **Preparedness for birth hampered;** Increased stress/anxiety due to changes in care	Changes to usual care structures, processes and care provision and the impact of these	*Altered care structures, processes, provision, and access*	
Feeling sad for partner; partner unable to bond with baby in first few days; partner attendance; partner restrictions; Feeling guilty as partner not present; Feeling angry as partner missing out	Restrictions on partner attendance and impact of this	*“It felt cruel” – restricting partners attendance*	**Theme 2 COVID related restrictions**
Alone or isolated (because of visiting or social distancing restrictions)	Feeling alone and isolated	*Restrictions in general: pros and cons*	
Visiting restrictions (in general); Self-restricting contact with others; Separated from baby; Forming close relationships with other women; Feeling cheated; **Missing out**	General visiting, access, and social restrictions; advantages and disadvantages		
Safety prioritised over experience; Staff safety prioritised over woman’s care; Precautions taken by MCPs; COVID testing/diagnosis	HCP precautionary and safety activities to protect against infection	N/A	**Theme 3 Infection prevention and risk**
Fear/worry of contracting COVID at visits; Hospital care less safe/as safe; Time of uncertainty; Benefit outweighs risk; Changing place of birth	Women’s thoughts and actions related to contracting/avoid contracting COVID		
Reduced support; Access to support; Loss of support; Good support; Seeking support; Sources of support; Breastfeeding support; Postpartum support; Mental health issues undetected	Support systems affected by COVID *(mostly negatively)*	*Psychosocial and information support*	**Theme 4 “*The lived reality*” – navigating support systems**
Media reports and influence; Information seeking; Information needs; Conflicting information; Help-seeking negatively affected; First time mother’s unique needs	Information sources and needs		
Finding solutions; Changing plans; Exploring alternatives; Being resilient and strong; Being in control; Preparing for birth; Comparing themselves to other women; Self-advocacy; Adapting to changes; Women’s recommendations to MCPs	How women addressed their support and information deficits	*Women’s solutioning*	
Advice from MCPs; Guidance expected from MCPs/needing reassurance; Good communication important; Challenges in accessing MCPs or services; **Birth options reduced;** Care from/communicating with MCPs; MCP more concerned about COVID than pregnancy issues	Good and poor interactions with HCPs and services	N/A	**Theme 5 Interactions with maternity services**
Feeling forgotten about; Feeling abandoned; **Being cared for stopped;** Unmet expectations; Awful experience; Poor care; Compassionate care; Disrespectful care	Care quality

**Table 4:** Theme development for maternity care providers’ views and experiences

Codes (reduced for illustrative purposes)	Descriptive themes	Analytical sub-themes	Analytical theme
Inadequate staff resources; Adequate staff resources; Access to safety resources; Staff training; Reduced capacity to provide care; Limited staff; Fear of illness impacting provision of care; Lack of personal contact as barrier to care provision; Language barriers; Lack of digital literacy as barrier to care; Clear communication as enabler; Telehealth enabling continued care	Staff resources, barriers, and enablers of care	*Capacity to provide care*	**Theme 1 Altered maternity care**
Reducing in-person care; Need for flexibility; Increased demand for homebirth; Restrictions impacting on women’s autonomy; Move to telehealth; Telehealth not optimal; **Increased medicalization of birth;** Rapid change; Changing protocols; Uncertainty in protocol; Certainty of protocol; Lack of informed decisions; COVID exacerbating inequalities; Racial inequalities; Inequalities in care; Minimal change to care	Changing provision of care, uncertainty, inequalities and continued need for care	*Altered care structures and provision*	
Positive change to workload; Increased staff workload; Increased staff need; Sense of collegiality; Impact on colleague relationships; Lack of support (colleagues/management); Feeling supported; **Conflicting professional beliefs; Inequalities in staff**	Impact on workload and relationships with colleagues	*Professional Impact*	**Theme 2 Professional and Personal Impact**
Increased personal burden; Increased financial burden; Emotional burden; Fear and anxiety; Two different worlds – in and outside hospital; Managing two lives – work and home; Sense of exclusion. Combative environment; A feeling of inevitability; Self as threat to others; COVID as threat to self	Burden, different worlds, and COVID as a personal threat	*Personal Impact*	
Future worries; Longer term impact on care provision; Longer term health outcomes	Future worries	N/A	**Theme 3 Broader structural impact**
COVID viewed as an opportunity; Improved provision of care; Improved health outcomes; Gaining a new perspective	COVID as an opportunity		

## Women’s views and experiences of maternity care during COVID-19

### Theme 1: Altered maternity care (women)

The theme of Altered maternity care (women) reflects how care changed for pregnant and postpartum women during the pandemic and the impact these changes had on their experiences of care. Thirty-one of the included studies contributed data to this theme. The two sub-themes of Altered care structures, processes, provision, and access, and Telehealth represent this analytical theme.

### Sub-theme 1.1: Altered care structures, processes, provision, and access

The pandemic resulted in considerable changes in how maternity care was structured, which affected care provision and access to care. Examples of these changes included: women attending clinics for care on their own, care transferred from in-person to virtual (sub-theme Telehealth), reduced choice for childbirth, inconsistent care, altered continuity of care (reduced and enhanced), schedules of fewer, postponed or cancelled antenatal or postnatal appointments, and altered maternity pathways based on COVID-19 test results [2, 35, 37–41, 44–47, 49–51, 55, 57, 58, 79]. For some women, the changes to maternity care provision were viewed positively. For example, antenatal care for some women involved a ‘…less busy waiting room’ [40, p.2019] and was reportedly more ‘streamlined, with reduced waiting times’ [2, p.6]. Reduced ‘inefficiencies’ were also noted, such as eliminating low-value visits, although this change was recognised as better serving women who had low-risk pregnancy [79]. In one study, where alterations to midwifery care were reportedly abrupt and continuous, most women felt they had received information on these changes that was ‘clear and timely’, which helped them adapt to and cope with the changes [44]. Overall, however, the alterations to maternity care were unsettling for women, causing increased stress, anxiety, worry, uncertainty, or dissatisfaction [38, 40, 41, 46, 49, 50, 52, 54–57]. Care provision for many women felt rushed, with limited time available to talk to maternity care providers or for normal checks, such as assessing blood pressure and performing scans, which were now not being done. As a result, women were left feeling anxious, overwhelmed, unsupported, or concerned [33, 37, 39, 46, 47, 52, 54, 55].

“Everything felt very rushed…. Nobody spent more than 10 minutes with me…. The entire time (in the hospital) I just felt rushed and alone.” [37, p.8]

Cancelled or postponed maternity care appointments, arising as a direct result of the pandemic, were commonly experienced by women [2, 34, 35, 43, 45–47, 49, 52]. This resulted in women feeling confused, worried, fearful, and abandoned [2, 39, 49–51]. Uncertainty and the ‘not knowing’ surrounding maternity care was also a considerable source of stress and anxiety for many women [2, 34, 39, 42, 44, 49, 56], resulting in some women entering the hospital for labour and childbirth already ‘tired’, ‘stressed’, ‘fearful’, and feeling ‘disillusioned’ [79]. Increased medicalisation of childbirth because of COVID-19 was also a concern for women. For example, having limited or no access to birthing pools as a birth option [39, 41, 47], ‘having alleged procedures (e.g., epidurals) forced on them’ or not being able to ‘have a normal delivery’ [39, p.25], and the ‘risk of a C-Section’ if diagnosed positive for COVID-19 [47] featured in women’s narratives. Pressures to be induced, and early discharge from the hospital after childbirth due to the pandemic were also cited as concerns for some women [37].

Altered systems of maternity care were identified in all studies contributing to this sub-theme, irrespective of country and birth setting, however, the extent of the changes appeared varied. For example, in Meaney’s international study [49], with participants mainly from the USA, UK, and Ireland (84%), restrictions on access to care varied across regions, and between hospitals within the same region, with some hospitals imposing less restrictions than others. These variations and inconsistencies in maternity care left women feeling frustrated and dissatisfied [37, 49, 79]. Continuity of care was also affected by the pandemic resulting in care continuity that was reduced, disjointed or non-existent [46]. Contrastingly, continuity of care in some regions was enhanced, especially between community and hospital settings [58]. Innovative and person-focused services which enabled home birth and continuity of services throughout the pandemic highlighted that supporting choice during COVID-19 was a possibility [41].

### Sub-theme 1.2 Telehealth

The sub-theme of Telehealth reflects women’s views and experiences of virtual rather than in-person maternity care during COVID-19. Telehealth was noted by women to confer some benefits; for example, avoiding travel time to the hospital or clinic for antenatal or postnatal appointments, overcoming long waiting times in clinics, being in the comfort of their homes and minimising exposure to risk of COVID-19 infection [52, 55, 74, 79]. The overarching narrative, however, was that telehealth was problematic for women and was favoured less than in-person care. Women were concerned that important information would be missed during telehealth consultations, especially regarding pregnancy or postpartum complications [33, 42, 45, 47, 52, 54, 55, 74]. This was especially evident in women who had pregnancy health or childbirth associated issues, such as hypertension, gestational diabetes, or perineal or caesarean wounds, and in women who had a previous stillbirth or miscarriage.

“And this telehealth situation, this monitoring from home, that’s a joke. It’s not going to work. How can you tell me that my C-section isn’t hurting when I’m telling you that it is hurting but you can’t see it” [74, p.4]

Online consultations for breastfeeding were described as ‘awkward’ and ineffective [2, 55, 75], and communication with maternity care providers was hampered as women found it difficult to develop a rapport during telehealth conversations [2, 45, 47, 74].

“And over the phone just doesn’t do it like. You don’t get the same, to look into somebody’s eyes and to trust them and for them to say, you’re okay” [2, p.14]

Women described feeling ‘embarrassed to talk about mental health concerns over the phone’ [47, p.3] such that telehealth was deemed inappropriate by women for discussing sensitive health issues. It also left some women feeling unprepared for birth, as in-person support mechanisms such as childbirth and parenting education classes were moved to the virtual space or were cancelled altogether [34, 74]. Telehealth also reduced the connection with maternity care providers that women considered important in pregnancy and postpartum care. The lack of in-person assessments resulted in many women feeling isolated, frightened, and anxious, and it led to mistrust amongst women; what women needed were in-person reassurances from their maternity care providers rather than virtual care that was largely perceived as inadequate or unsatisfactory [2, 34, 39, 46–48, 52, 74].

### Theme 2: COVID related restrictions

Eighteen studies contributed data on women’s views and experiences of pandemic imposed restrictions. The synthesis of these data is represented in two sub-themes; ‘It felt cruel’—restricting partners attendance and Restrictions in general: pros and cons.

Sub-theme 2.1: ‘It felt cruel’ – restricting partners attendance

The degrees to which COVID-related restrictions were imposed on women’s partners or primary support person varied. For example, for some women, partners could remain throughout the birth and postpartum periods [52, 55], or throughout the birth and for a short period afterwards [38, 58]. For others, partner presence was not permitted at all during labour and childbirth [36, 44], or permitted only when women were deemed to be in ‘active’ labour [2, 76]. In almost all studies, however, participants indicated that partners were prohibited from attending antenatal appointments or routine postnatal follow-up visits [34, 40, 42, 43, 46, 47, 58]. Restrictions on partner attendance throughout the maternity care continuum evoked a wide array of emotions for women. These emotions ranged from feelings of guilt [38, 44, 55], anger [38, 49], emptiness [44], sadness [2, 44, 46, 49, 58], bitterness [44], anxiety or stress [39, 42, 46, 49, 55], fear [39, 52], worry or concern [38, 39, 43, 47, 52, 58], and disappointment [52]. Significantly, women expressed intense feelings of being alone, isolated, and lonely because of the restrictions [2, 37–39, 56, 76]. It was clear that women had a strong desire or ‘needed’ to have their partner present throughout the maternity care experience, even for those simple supportive and reassuring gestures “…. like, to hold your hand, or to tell you that it would be ok” [2, p. 15]. Imposed partner separation at the time of birth was extremely distressing for women and resulted in a labour and birth that felt ‘unfulfilled’ [44]. Concerningly, restrictions on partner attendance evoked intense emotions which could potentially have lasting effects:

“I’m so angry that neither I, nor [name], will ever get that day back. I will never be able to correct it or make it a better experience … it felt cruel [38, p.1]

“… denying my husband, the right to be there, or me the support he provides is a disgusting standard of care which will have lifelong effects” [39, p.6]

### Sub-theme 2.2: Restrictions in general: pros and cons

This sub-theme reflects women’s experiences and views of restrictions beyond those related to restrictions on partner attendance. Restrictions, based on hospital policy, because of being positive for COVID-19 or restrictions imposed when babies were admitted to the neonatal intensive care unit (NICU) appeared especially harrowing and distressing for women [36, 37, 44, 45, 58, 75]. Being separated from their baby left women feeling like they had done something wrong [75] or that they had ‘abandoned’ their baby [45]. New visiting rules meant parents were only allowed to visit their baby for a very short period during the day [58] or not at all [45]. This separation resulted in women being unable to touch, feel, cuddle, or smell their babies, and, in recounting their experience, women’s voices ‘trembled with tears alternating to silences’ [44, p.8]. Isolation and separation from friends and the wider family also affected women, albeit in various ways. Women expressed disappointment that they were not able to engage in traditional pregnancy rituals, share their pregnancy journey with family and friends or celebrate their pregnancy and birth with others, resulting in feelings of missing out or of loneliness [34, 42, 49–51, 55].

“It’s made it definitely a more somber experience and it has been difficult to be excited because you can’t share it with people.” [42, p.5]

Attending or being in hospital alone without visits from friends and family was difficult for many women [37, 38, 40, 41, 43, 44, 56]. The separation and isolation from wider support networks resulted in women experiencing fear [36, 47] or feeling ‘cut-off’ [41], without freedom to move around [49] as ‘you are in a room, you can’t go out and you’ve got to stay between those four walls’ [44, p.8]. This isolation and loneliness continued for many women into the postnatal period, especially as women were denied opportunities to introduce their new baby to loved ones. Lack of interactions with family and friends following childbirth affected women’s mood negatively. Women recounted feeling ‘overwhelmed’ or anxious without having the help from family and friends in caring for their baby that they would otherwise have had [51, 55, 56, 75]. Contrastingly, the wider visiting restrictions in hospital beyond partner visiting, and when women returned home, were a positive experience for some women. Women attributed reduced visiting in the postnatal ward as providing extra space and time to bond with their babies [2, 40]. Quieter postnatal wards facilitated a private space for women to establish breastfeeding more comfortably [2, 40] and women drew comfort from the ‘peace and quietness’ offered by less crowded postnatal wards.

“It is a lot quieter, more time to adjust and try to get a hang of breastfeeding without an audience” [40, p.220]

Visiting restrictions and being isolated encouraged women to form close relationships with other women in a similar situation [2, 44]. On returning home from the hospital, because they could ‘politely decline people coming over’ [56, p.3], this enabled some women to enjoy quiet, precious time as a family unit.

## Theme 3: Infection prevention and risk

Narratives around risks associated with contracting COVID-19, as well as infection prevention were evident in 29 of the included studies. Many women, perceiving the maternity care facility as a source of infection risk, were fearful, worried, and wary of visiting the facility for fear of contracting the virus [34–36, 39, 41, 43, 44, 47, 48, 50, 52, 55, 57, 75, 77]. Due to this fear, many women described taking proactive measures to minimise the risk of potential infection. Examples included changing their hospital to attend a hospital that cared only for pregnant women [53], considering or opting for a home birth [34, 39, 41, 44, 46, 47, 76], missing, avoiding, or postponing hospital care visits [2, 35, 41, 43, 47, 77, 78] and adhering rigidly to infection control measures [48, 55].

“We’re more concerned about whether we came into contact with anything in the hospital” [75, p. 1275]

The interplay between balancing fear of contracting COVID-19 and the risk of not attending for care was a source of emotional conflict for some women, presenting them with a challenging dilemma [39, 46, 77]. Uncertainty about virus transmission, and what impact contracting COVID-19 might have on their pregnancy health and maternity care throughout the antenatal, intrapartum, and postnatal continuum was also evident in women’s narratives, especially during the early stages of the pandemic [34, 43, 44, 49, 51, 74]. Some women, however, while accepting that infection control measures were implemented to safeguard against virus transmission, felt that the birth experience was being disregarded, compromised, or viewed secondary to infection control as a result [33, 37, 39, 41].

“At present, all that matters is keeping the baby safe and keeping the mother well enough to give birth, disregarding the humanized dimensions of pregnancy, birth care, and the lived experience of the birthing mother” [41, p.17]

Women, however, were appreciative of efforts in maternity care settings to minimise virus transmission and felt reassured by these. Measures described by women included separate areas or entrances for pregnant women attending for antenatal care [52], high levels of hygiene measures or social distancing precautions [2, 34, 40, 46, 52], temperature checking or symptom screening on arrival [2, 34, 45, 75], keeping women up to date when protocols changed [58], and staff use of PPE [44].

## Theme 4: “The lived reality” – navigating support systems

This theme reflects the reality of navigating support during pregnancy and the postpartum period, and the activities that women undertook to address challenges associated with this. Twenty-three studies contributed data to this theme, with findings reflected across the two sub-themes of Information and psychosocial support and Women’s solutioning.

### Sub-theme 4.1: Information and psychosocial support

Navigating information and psychosocial support during the pandemic appeared especially challenging for women. Women experienced navigation difficulties for various reasons and due to different causes. Information support, for example, was affected by a lack of consistent messaging, conflicting information or a lack of clear guidance surrounding the virus and how this affected women’s care, both during pregnancy and postnatally [37, 39, 43, 46, 49, 51, 53, 56].

“One doctor would say one thing and then the next would say another.” [37, p.6]

This resulted in women feeling lost, confused, or helpless [39, 46, 55]. Having trustworthy information became a key concern for women and many women recounted a need for further information [36, 42, 47, 49, 52]. In navigating their information needs and support, many women resorted to alternative sources, mainly social media, television, and online sources, as well as friends and, in some cases, government sources [34, 38–40, 44, 47, 50, 52, 53, 55, 56]. The use of these alternative sources was, however, problematic as women recognised that such sources can be unreliable or were a causal source of stress and fear [44, 47, 50, 53, 56]. Although some women were sent information by their maternity care providers, many women recounted a desire for greater levels of official communication from the hospital or their care providers:

“…and I think probably one thing that maybe could be improved is just that extra information of what you are doing with the COVID stuff in terms of precautions, what it's going to look like when I come in to have bubs, just what to expect.” [34, p.6]

Navigating psychosocial support also featured considerably in women’s narratives. Although some women recounted receiving good support from maternity care providers [40, 55, 58], for many women this type of support was significantly diminished, leaving women finding their own way ‘as there was nobody else to help’ [38, p.1]. Furthermore, because of reduced psychosocial support, many women were left feeling concerned that mental health problems would go unnoticed or left not knowing where to seek support should problems arise for them [2, 47, 52]. Women viewed dedicated formal support from maternity care providers as essential for their psychosocial wellbeing. When this was lacking or diminished, many women turned to and relied heavily on informal supports such as family and friends as a substitute [2, 34, 36, 44, 49]. Concurrently, however, informal supports such as peer, family, and other social supports (e.g., mother and baby groups), were no longer available for many women [2, 34, 37, 41, 42, 44, 45, 49, 51, 55, 56, 74] leaving women with limited or no support at all. Lack of breastfeeding support, especially in-person support, also featured heavily in women’s narratives [2, 47, 52, 54, 55]. This left many women having to work through issues on their own which was a source of disappointment for them:

“I was struggling breastfeeding. I would have gone to breastfeeding group, but that’s been cancelled…. I was in pain and I felt let down” [52, p. 20]

### Sub-theme 4.2: Women’s solutioning

Women, in navigating their maternity experience during the pandemic, self-implemented solutions as a means of coping, including adjusting their plans or exploring other options for care [39, 47, 56]. Many women demonstrated resilience by trying to ‘forget’ the past and build a new normal for themselves [44], or by trusting their own judgement and instincts [51]. Women were proactive in preparing themselves for birth, with some describing how they actively sought out online classes in the absence of formal professional supports [34, 41]. Some women went to extreme measures to ensure they were prepared for all eventualities such as buying their “own IV fluids and cannulas and respiratory equipment, so that if the baby wasn’t breathing then we could do something about it” [56]. Women also compared themselves to other women, and drew comfort from each other:

“And you know if you were kind of just worried, but you were able to talk to each other. And just comfort each other.” [2, p.18]

Women spoke of advocating for themselves to achieve the maternity care they desired or needed, amidst the constant changes occurring within maternity systems [2, 37, 39, 46, 56]. For example, some women took the decision to book for an induction of labour or elective caesarean to minimise uncertainty and gain ‘some control over a situation that was uncontrollable’ [2, p.17]. While women described not wishing to ‘fight’ for what they required, many felt that they had no choice but to do so [39, 46, 56].

“I am forced to continually fight to be seen and have to reiterate my situation and reasoning over and over …. and now I have no choice but to advocate for myself but it has been very difficult.” [46, p.5]

Other women, alternatively, found themselves simply accepting of and being adaptive to the present situation which helped them feel prepared [2, 44, 51, 76].

## Theme 5: Interactions with maternity services

The theme of Interactions with maternity care providers reflects women’s experiences, both positive and negative, of their engagement with the maternity services and maternity care providers. Fifteen studies contributed data to this theme. Women recounted being either unable to contact, or experienced fewer interactions with, their care providers antenatally, while in the hospital, or during the postnatal period [2, 36, 37, 39, 41, 43–47, 49, 52, 53, 74, 75]. This led women, in general, to view their care as inadequate, sub-par, disrespectful or of poorer quality. Women attributed these altered interactions to care providers trying to limit their exposure [49, 74], viewing women as a potential infection risk [46], or that maternity care providers were more concerned about COVID-19 itself than pregnancy related issues [39, 46, 47, 52, 55, 56, 77].

“…The education given by the OB has dramatically shifted from normal pregnancy concerns to 95% about coronavirus. I feel like my questions about non-COVID issues are getting overlooked.” [46, p.4]

This ‘hands-off’ style of care resulted in a ‘colder birthing experience for women’ [37, p.8] or left women feeling neglected by their care providers [39, 46, 49, 75]. Some women, however, experienced supportive and reassuring care via non-verbal body language, gestures, and looks where they “…could tell from their eyes that they were taking care of you” [44, p.6]. In addition to fewer and reduced quality interactions, interaction settings also changed for women due to the pandemic; for example, having to attend a different area of the hospital or attending a different hospital or clinic for care [47, 48, 50, 53]. For some women, this caused confusion as to whether they would have timely access to care [34] or presented transport challenges that did not exist previously [77]. How maternity care providers would treat women should they be diagnosed with COVID-19 was a further concern for some women [44, 47]. Welcoming and non-judgemental verbal language was important to women; however, some women who were positive for COVID-19 infection experienced what they perceived as nonprofessional and inappropriate interactions:

On several occasions they told me ‘Stay away, stay away, keep the 1-meter distance, go to that corner in the lift…. When they came in the room to wake me up at 6am they used to open the door shouting ‘masks!’ [44, p.8]

Those who were positive for COVID-19 used intense language in describing their overall birth experience including words such as ‘traumatic’, ‘tragic’, ‘difficult’, ‘strenuous’, ‘sad’, ‘disheartening’, ‘terrible’, ‘negative’, ‘odd’ and ‘unfortunate’ and used the analogies of being in a ‘nightmare’ or a ‘war’ to convey perceptions of their experience. Women also spoke of unmet expectations arising from interactions with their maternity care providers [39–42, 44, 56]. This was a source of disappointment for some women and affected their ability to prepare properly for the arrival of their new baby [44, 56]. Women offered suggestions as to how unmet expectations should and could be addressed, including: a case-by-case easing of restrictions where there were extenuating circumstances such as when women or babies experienced complications [40], better dissemination of hospital COVID-19 policies as well as enhanced communication between women and care providers [55, 75], and COVID-19 testing early in the admission process [40].

## Maternity care providers’ views and experiences of maternity care during COVID-19

### Theme 6: Altered Maternity Care (providers)

The theme of Altered maternity care represents narratives from maternity care providers on how the provision of care substantially changed during the pandemic and the impact that this had on their capacity to provide appropriate and effective care. Twenty-two studies contributed data to this theme. The two sub-themes of Altered care structures and provision and Capacity to provide care were identified within this analytical theme.

Sub-theme 6.1: Altered care structures and provision.

Across all settings, the provision of care had substantially changed for maternity care providers. There was a focus on reducing in-person appointments with a move to virtual or telephone appointments, where possible, which required maternity care providers to be innovative and adaptive in identifying alternative ways to provide care [17, 64, 66, 71, 72]. Maternity care providers also described a reduction in the numbers of women accessing certain types of maternity services (e.g., inpatient antenatal care, postnatal clinics, and infant immunisation appointments) due to concerns about attending in-person [72, 78], while, concurrently, the demand for midwifery care at home and homebirth in some settings had increased [59, 69, 71, 72]. A feeling of uncertainty was dominant in maternity care providers’ narratives, largely influenced by the rapid speed with which care protocols were changing [17, 60–63, 65, 68, 70, 72, 74]. Constant change and inconsistencies across settings often led to confusion and differences in interpretation [66, 70, 73]. Some expressed that this may have negatively influenced the care that was provided as it was unclear if the new care protocols were sufficiently evidence-based [69, 70, 76].

“Departmental protocols…were changing rapidly, leading to confusion and unclear interpretation by staff members… variation in practice and misinterpretation of guidance were expressed…especially where limited evidence is available” [70, p.1125].

Many maternity care providers, however, acknowledged that such uncertainty lessened over time as clear national guidelines became established and implemented, and the communication around care protocols improved [17, 62, 65, 66, 73].

Many maternity care providers also believed that the pandemic exacerbated existing inequalities in maternity care. The closing of some services or moving maternity appointments to virtual or tele settings were viewed as having a greater impact on at-risk communities as this group were less likely to be able to access these types of alternative services [61, 64, 67, 69, 71, 72]. Furthermore, some maternity care providers held the view that other providers were instigating racist or sexist practices based on inappropriate or misconstrued beliefs around the risk of COVID-19 in certain population groups, subsequently exacerbating the existing challenges that pregnant or postpartum women may already be facing [61, 68, 74].

“Restrictions and regulations in the time of COVID-19 have allowed for a resurgence of the racist and sexist policies…Black women’s bodies have continued to be seen as risky…leading to a lack of care and touch that continues to put Black birthing people in danger” [68, p7).

Some providers also held a belief that other maternity care providers were using pandemic associated changes as an excuse for inadequate care [69, 75].

“COVID has also given practitioners justifications for many unnecessary and excessive practices; when negligence is not the issue, increased intervention is” [69, p3].

Some practice changes, however, were viewed as having a positive impact on care due to fewer women attending services which increased the time available to spend with each woman and which facilitated a more efficient service [73]. Despite the pandemic and the associated changes to care structures and provision, maternity care providers were emphatic in describing that the need for maternity care did not stop as “women’s health care needs …could not be put on pause” [15, p.3]; something which was considered unique to maternity care provision [15, 60, 62, 63, 69].

### Sub-theme 6.2: Capacity to provide care

Most maternity care providers felt that the changes due to COVID-19 had resulted in a reduced capacity to provide safe and effective care. A lack of access to adequate resources, such as PPE and training on safe practices, left maternity care providers feeling that they were not adequately protected [17, 63, 65, 66, 70, 74, 77]. As a result, providers limited their interactions with women during pregnancy or during the birthing process as they feared being infected and/or acting as a vector of infection [65, 75, 77].

“To decrease the risk of transmission, we usually compromise the routine antenatal care service. For instance, we may not perform physical examination or draw blood, even if necessary” [77, p.4]

COVID-19 related restrictions, such as reduced in-person appointments, cancelled support groups, and reduced numbers of maternity care providers in the birthing suite, were viewed as barriers to care [17, 59, 64, 68, 69, 71, 74, 75]. Personal contact was viewed as an essential part of maternity care, especially by midwives. Having to limit this element of their care was perceived as having a negative impact on their capacity to provide care, with some viewing it as a “dehumanization of childbirth” [65, p4]; especially for certain activities such as lactation support, where personal contact was considered critically important.

“Virtually the screen is small, I’m at the mercy of the person holding the phone…I have to verbally direct the mom over the phone, and many interrupt[ion]s on both sides of the conversations” [71, p.265].

A move to telehealth was viewed positively by some as it enabled the continuation of care in a safe environment and allowed for a more responsive approach to care in some circumstances. However, telehealth was described as having limitations. Due to the lack of personal contact, it was viewed as hampering the ability to build a trusting relationship with women which impacted on providing effective care [64, 68, 69, 71].

“Over the telephone, it is harder to read all the non-verbal cues as you would in an in-person counselling session” [71, p.265]

Furthermore, language barriers and insufficient access to digital resources or a lack of digital literacy sometimes hindered the provision of care in this mode. This was an issue especially for maternity care providers in low- and middle-income countries, or for disadvantaged populations in high income countries [64, 67, 71].

“One of the biggest challenges reported was poor internet connection and/or regular interruptions in connectivity. This was a global problem reported by providers from both LMICs and HICs” [64, p.8]

## Theme 7: Professional and Personal Impact

The theme of Professional and Personal Impact describes the impact of the COVID-19 pandemic, as expressed by maternity care providers, on their professional careers and their personal lives. Nineteen studies contributed data to this theme. The two sub-themes of Professional impact and Personal burden were identified within this analytical theme.

### Sub-theme 7.1: Professional Impact

Professional impact was predominantly negative for maternity care providers. Staff shortages [65, 73], additional tasks due to new care practices [62, 66, 68, 69], and longer and more frequent appointments as pregnant and postpartum women required more interaction due to anxiety and uncertainty, all contributed to an increased workload [17, 59, 64, 68, 73]. For maternity care providers working outside the traditional hospital system, such as independent community midwives, their workload substantially increased as the demand for a non-hospital birth experience increased [17]. There was one ‘lone voice’, however, that expressed a more positive experience in relation to workload whereby previous practices such as ‘overbooking of inductions’ ceased and ‘caesarean section lists reduced’ as part of pandemic efforts to strictly manage numbers in the hospital [70]. A further ‘silver lining’ described by maternity care providers from the experience of working on the frontline during the pandemic was ‘the bond’ it created amongst providers as they worked together [15, 61, 70]. The uncertainty and chaos introduced by the pandemic helped to build relationships as maternity care providers supported one another in providing care during these unprecedented times [61, 62, 66, 74]. In some ways, life in a maternity care setting acted as a bubble to the outside world where it was perceived that people were cut off from each other to a greater extent, and the hospital was associated with a greater sense of calm and camaraderie [59, 60].

“It was ‘on the frontlines’ that I felt the most the distant from the pandemic itself. I felt guilty responding to messages from family and old friends, those not in medicine but trapped in their homes by an invisible enemy ravaging their cities and towns” [60, p.2]

Contrastingly, in some settings, divisions emerged as particular professions pitted themselves against others, for example, midwives and doctors [65, 69, 74, 76].

“The pandemic has impacted the medical culture in Puerto Rico, emboldening doctors to ‘protect’ their ‘domain’… medical professionals have taken to social media…gone on the news to argue that the absolute safest place to give birth is the hospital” [69, p.5]

Different professional beliefs or perceptions about status in the ‘hospital hierarchy’ also contributed to these divides. Altman and colleagues described how nurses working in perinatal settings felt that they were expected to take additional risks to those of other staff members, including consultants [74]. This contributed to a feeling that nurses were expendable and not valued for their contribution to care. There was an evident divide between frontline maternity care providers and management. Inadequate communication about changing protocols and a perceived lack of consideration of staff needs resulted in many care providers feeling abandoned by management [59, 63, 69, 72, 74, 76, 77]. This added to the feeling of uncertainty and was an additional emotional burden to burdens that were already present.

“Nurse participants described ...wanting more compassion and respect from hospital administration…a need to be seen as an individual who is being placed at risk” [74, p.6]

### Sub-theme 7.2: Personal Burden

The pandemic presented a personal burden for all maternity care providers “including issues that affected personal health and well-being; challenges with family and parenting; and mental health concerns, stress, finances and loss of income. This was universal regardless of personal circumstances, type of practice, years in practice or geographical area” [73, p.4]. COVID-19 was considered a significant threat, and due to a fear of acting as a vector, to either patients or family members, maternity care providers isolated themselves from or interacted less with their families [17, 59, 61, 65, 66, 68, 69, 72, 73, 77, 78]. This carried a significant emotional burden as they dealt with the uncertainty and anxiety of the pandemic in isolation [63, 66, 68, 73]. This self-imposed isolation led to concerns about the wellbeing of dependent family members where maternity care providers questioned their parenting capacity; this further added to their experience of emotional burden, a burden which was further compounded by the feeling of abandonment by management [69, 74]. Maternity care providers described making substantial sacrifices to ensure the continuation of care but felt that little recognition was being given for this [62, 74]. Some maternity care providers also spoke about the financial burden that they were experiencing because of the pandemic, which added to existing emotional burden [64, 68, 73]. As the demand for certain services reduced, this had a negative impact for those working independently, such as community midwives, resulting in reduced income which was worrisome. Others spoke about needing to adapt their mode of care, such as moving to telehealth, but not being adequately reimbursed.

“Respondents themselves faced financial burdens from the use of telemedicine on two levels: not being able to afford the equipment and lack of reimbursement…for costs they incurred while providing telemedicine (including the telehealth consultation itself and its associated internet/phone/data costs)” [64, p.9]

## Theme 8: Broader structural impact

While much of the focus in the studies included in this QES was on the immediate implications for maternity care providers, important findings were also identified in relation to the perceived broader impact of the pandemic on maternity care, with data from 10 studies contributing to this theme. Some maternity care providers were worried about the impact of restrictions on the future health outcomes of parents and babies. They believed that the restrictions led to reduced care and support being provided, which they believed would have a negative impact on future health:

“[the] lack of time and staff will lead to mothers and babies going home with very little feeding support or knowledge which will have a short- and long-term impact on their health and ability to deal with infections” [72, p.7]

There were also concerns that some of the changes introduced due to COVID-19 would be retained in the post-pandemic era as they were viewed as economically beneficial by management, or for reasons of self-interest by certain providers who wished to minimise in-person care. The potential retention of such changes was viewed as being detrimental for future maternity care [59, 69, 76].

“I feel management will see the changes made i.e. shorter inpatient stay, increased VMS (Visiting Midwifery Service) personnel as economically beneficial and it will be difficult to revert back” [59, p.8]

While maternity care providers largely experienced and described the COVID-19 pandemic as negative, there were some who viewed the situation as an opportunity [15, 59, 61, 66, 68, 69, 73, 76, 78]. Restricting visitor numbers on post-natal wards was viewed as a positive change as it helped to better establish feeding routines, while incorporating telehealth into practice offered greater accessibility to certain groups [15, 59, 69, 73]. It was thus hoped that some of these changes could be retained, to some extent, as restrictions eased. It was also hoped that the pandemic had drawn attention to longstanding gaps in maternity care, with racial and socioeconomic inequalities highlighted [61, 76, 78].

“Going through this dual pandemic of COVID-19 and systemic racism is exhausting, to say the least. I reminisce of the “before” times but it’s been long overdue for the veil to be lifted - and for that reason I am grateful for the chaos” [61, p.2]

COVID-19 related restrictions also prompted maternity care providers to take a different perspective of their role and how best they could support parents [59, 61, 66, 68]. For example, there appeared to be a greater focus on empowering parents to care for themselves and advocate for their birth preferences which was viewed as a more positive approach to care [68, 69, 76].

“With telehealth and remote appointments there is a lot of emphasis on self-care and being aware of your health - it is empowering for women” [69, p.5]

Due to this change in perspective, some maternity care providers considered the pandemic as a period of growth as they gained confidence in their role by successfully addressing uncertain situations and were looking for further opportunities for development [59, 61, 66, 68].

## Confidence in the review’s findings: GRADE-CERQual

Twenty-seven discrete findings were subjected to GRADE-CERQual confidence assessments; of which 16 were from the synthesis of women’s data and 11 were from the synthesis of maternity care providers’ data. Table 5 presents the summary results. Additional File 3 presents the detailed Evidence Profile and rationale for judgements in each of GRADE-CERQual’s four components. Overall, confidence in the review’s findings was either high (*n* = 14 findings) or moderate (*n* = 13 findings), providing reassurance for the applicability of the findings for informing clinical practice and care. Most of the downgrading in confidence related to the adequacy of the data, whereby the richness, depth and quantity of data contributing to a finding was affected by the diversity of study designs, especially survey design. Importantly, all findings were judged to have no, very minor or minor concerns on coherence. This means that the extent of support for each review finding from the underlying data was high; in other words, the individual included studies were reporting similar information in terms of how women and providers experienced maternity care during this time. Some minor concerns were applied to the relevance of some findings, which relates to how closely the inclusion criteria of the included studies mirrors that of the review question. Although all studies met the aim and inclusion criteria of our QES, due to the specific focus that some studies took, we judged this to have a potential impact on relevance. For example, studies that focused on women who were COVID-19 positive only [44], women who were undergoing prenatal genetic testing [43], or women’s experiences of online support classes exclusively [41].

**Table 5:** GRADE-CerQUAL summary results

Finding	Contributing records	Methodological limitations	Coherence	Adequacy	Relevance	Overall confidence
**Analytical theme 1: Altered Maternity Care (women)**						
Alterations to maternity care, overall, were unsettling for women, causing increased stress, anxiety, worry, uncertainty, or dissatisfaction	38, 40, 41, 46, 49, 50, 52, 54–56, 57	Minor concerns	No or very minor concerns	Minor concerns	Minor concerns	**High**
Uncertainty and inconsistencies surrounding maternity care were a considerable source of stress, anxiety, frustration, and dissatisfaction for women	2, 34, 37, 39, 42, 44, 49, 56, 79	Minor concerns	Minor concerns	Minor concerns	Minor concerns	**High**
Cancelled or postponed maternity care appointments were commonly experienced leaving women feeling confused, worried, fearful, and abandoned	2, 34, 35, 39, 43, 45–47, 49–52	Minor concerns	No or very minor concerns	Moderate concerns	Minor concerns	**Moderate**
Telehealth was noted to confer some benefits; overall, however, telehealth was problematic for women and was favoured less than in-person care	2, 33, 34, 39, 42, 45–48, 52, 54, 55, 74, 75, 79	Minor concerns	No or very minor concerns	Minor concerns	Minor concerns	**High**
**Analytical theme 2: COVID related restrictions**						
Restrictions on partner attendance throughout the maternity care continuum evoked a wide array of emotions for women including intense feelings of being alone, isolated, and lonely	2, 38–39, 42–44, 46, 47, 49, 52, 55, 56, 58, 76	Minor concerns	No or very minor concerns	Moderate concerns	Minor concerns	**Moderate**
Isolation and separation from friends and the wider family affected women in various ways (disappointment, loneliness, fear, anxiety, overwhelmed), although the wider visiting restrictions in hospital beyond partner visiting, was a positive experience for some women	2, 34, 36–38, 40–44, 47, 49, 50, 51, 55, 56	Minor concerns	Minor concerns	Moderate concerns	Minor concerns	**Moderate**
**Analytical theme 3: Infection prevention and risk**						
Fear of contracting COVID-19 was prominent for women, with many fearful, worried, and wary of visiting the maternity care facility for fear of contracting the virus	34–36, 39, 41, 43, 44, 47, 48, 50, 52, 55, 57, 75, 77	Minor concerns	No or very minor concerns	Moderate concerns	Minor concerns	**Moderate**
The interplay between balancing fear of contracting COVID-19 and the risk of not attending for care was a source of emotional conflict	39, 46, 77	No or very minor concerns	No or very minor concerns	Moderate concerns	Minor concerns	**High**
Women were complementary and appreciative of efforts in maternity care settings to minimise virus transmission and felt reassured by these	2, 34, 40, 44–46, 52, 58, 75	Minor concerns	No or very minor concerns	Moderate concerns	Minor concerns	**Moderate**
**Analytical theme 4:* “The lived reality” –* navigating support systems**						
Information support was affected by a lack of consistent messaging, conflicting information or a lack of clear guidance surrounding the virus and how this affected women’s care, which left women feeling lost, confused, or helpless	37, 39, 42, 46, 49, 51, 53, 55, 56	Minor concerns	No or very minor concerns	Moderate concerns	Minor concerns	**Moderate**
Women viewed dedicated formal support from maternity care professionals as essential for their psychosocial wellbeing; however, these supports were largely diminished or lacking	2, 34, 36, 37, 41, 42, 44, 45, 49, 51, 55, 56, 74	Minor concerns	No or very minor concerns	No or very minor concerns	Minor concerns	**High**
In navigating information support, many women resorted to alternative sources, mainly social media, television, and online sources, as well as friends, although women recognised that these alternative sources could be unreliable which caused stress and fear	34, 38–40, 44, 47, 50, 52, 53, 55, 56	Minor concerns	No or very minor concerns	Minor concerns	No or very minor concerns	**High**
Women self-implemented solutions as a means of coping, including adjusting their plans, exploring other options for care or self-advocating to achieve the maternity care they desired or needed	2, 37, 39, 47, 56	Minor concerns	Minor concerns	Moderate concerns	No or very minor concerns	**Moderate**
**Analytical theme 5: Interactions with maternity services**						
Women recounted being unable to contact or experienced fewer interactions with their care providers which led women, in general, to view their care as inadequate, sub-par, disrespectful or of poorer quality	2, 36, 37, 39, 41, 43–47, 49, 52, 53, 74, 75	Minor concerns	No or very minor concerns	Moderate concerns	Minor concerns	**Moderate**
Some women who were positive for COVID-19 experienced what they perceived as nonprofessional and inappropriate interactions	39, 44, 46, 47, 52, 55, 56, 77	Minor concerns	No or very minor concerns	Minor concerns	Minor concerns	**High**
Women experienced unmet expectations arising from interactions with their maternity care providers source which affected their ability to prepare properly for the arrival of their new baby	39, 40–42, 44, 56	Minor concerns	No or very minor concerns	Moderate concerns	Minor concerns	**Moderate**
**Analytical theme 6: Altered Maternity Care (maternity care providers)**						
A feeling of uncertainty was dominant across providers, largely influenced by the uncertainty surrounding care protocols and the speed at which these changed, although this uncertainty lessened over time as national guidelines became available and communication of care protocols improved	17, 60–63, 65, 66, 68, 70, 72–74	Minor concerns	No or very minor concerns	No or very minor concerns	No or very minor concerns	**High**
The pandemic was considered to have exacerbated existing inequalities in maternity care	61, 64,67–69, 71, 72, 74	Minor concerns	No or very minor concerns	No or very minor concerns	Minor concerns	**High**
The lack of access to adequate resources and training on safe practices resulted in providers limiting their interactions with women as they feared being infected and/or acting as a vector of infection	17, 63, 65, 66, 70, 74, 77	Minor concerns	No or very minor concerns	Minor concerns	No or very minor concerns	**High**
A move to telehealth was viewed positively by some as it enabled continuation of care in a safe environment, although it was not without its limitations	64, 67–69, 71	Minor concerns	Minor concerns	Moderate concerns	Minor concerns	**Moderate**
**Analytical theme 7: Professional and Personal Impact**						
The pandemic had resulted in an increased workload for maternity care providers, due to staff shortages, additional tasks, and more frequent and longer appointments	17, 59, 62, 64–66, 68, 69, 73	Minor concerns	Moderate concerns	Minor concerns	Minor concerns	**Moderate**
Relationships with colleagues improved as maternity care providers supported each other through the uncertainty, although the pandemic also deepened divisions due to perceived staff hierarchies and disconnect between management and providers involved in direct care	15, 59–63, 65, 66, 69, 70, 72, 74, 76, 77	Moderate concerns	Minor concerns	Minor concerns	No or very minor concerns	**Moderate**
Maternity care providers isolated themselves or restricted their interactions with family due to a fear of transmitting the virus to others, which carried a significant emotional burden	17, 59, 61, 65, 66, 68, 69, 73, 77, 78	Minor concerns	No or very minor concerns	Minor concerns	Minor concerns	**High**
The pandemic had a negative financial impact for some providers due to reduced service demand and inadequate reimbursement for alternative services, such as telehealth	64, 68, 73	No or very minor concerns	No or very minor concerns	Moderate concerns	Moderate concerns	**Moderate**
**Analytical theme 8: Broader structural impact**						
Restrictions were considered by some to have a negative impact on future health outcomes for parents and babies, while others worried that certain restrictions would be retained, and these would negatively influence future maternity care	59, 69, 72, 76	Moderate concerns	No or very minor concerns	Minor concerns	No or very minor concerns	**High**
The pandemic was viewed as an opportunity to improve maternity care, including addressing inequalities, and implementing changes that supported parents and their babies	15, 59, 61, 69, 73, 76, 78	Moderate concerns	No or very minor concerns	No or very minor concerns	Minor concerns	**High**
The pandemic prompted some maternity care providers to take a different perspective of their role and considered it an opportunity for professional growth	59, 61, 66, 68, 69, 76	Minor concerns	No or very minor concerns	Minor concerns	Minor concerns	**High**

## Discussion

This comprehensive QES based on the inclusion of 50 records of primary studies, provides insight and understanding of how women and maternity care providers experienced maternity care during the COVID-19 pandemic. Reassuringly, all 27 discrete findings were judged to have either high or moderate confidence. The narratives in many of the included studies were similar in content, even though contexts differed. For example, irrespective of country, region or maternity care setting, maternity care for both women and maternity care providers altered significantly and rapidly because of the pandemic. The move towards telehealth brought some benefits, although, more often, challenges. Digitalisation is advancing internationally, including in health. There are currently more than 10,000 computerised programs or m-health apps for promoting positive mental health [81]. Digital health can be beneficial for self-care and self-management activities; however, the findings of our QES point to the ineffectiveness of telehealth as a replacement for in-person care. As the pandemic continues, and new variants emerge, innovative ways to ensure in-person care can continue, are required.

The QES findings that maternity care during COVID-19 evoked an array of adverse and negative emotions for both women and maternity care providers are significant as they provide insight into possible future health challenges that women and maternity care providers may experience. Future mental wellness, healthy maternal-infant bonding, coping, resilience and burn-out are examples that warrant careful consideration. Unlike distress during other life periods, the perinatal period appears particularly critical, with negative psychological or psychosocial events in pregnancy, birth and early life appearing to have exaggerated life-long consequences, not only for mothers, but also for their infants. In a systematic review of 122 studies on maternal postpartum depression and the consequences for infant health up to 6 years of age, infants of mothers experiencing postpartum depression were found to have poorer weight gain in infancy, a greater proportion of child illnesses, increased infant night-time awakenings, and other problematic sleep patterns [82]. The review also found a negative association between maternal postpartum depressive symptoms and cognitive development in children, lower child social engagement, higher fear scores and degrees of child anxiety and negative behaviours, and lower mother-infant bonding [82]. Social isolation and a lack of social support, consequences of the pandemic, have been described as risk factors for increased perinatal adversity [83] and can lead to reduced maternal quality of life, and adverse physical, behavioural and development health in babies [84]. Furthermore, diminished care, such as limited or poor attendance for antenatal care increases the risk for perinatal mortality, preterm birth, and low birth weight babies [85]. These findings emphasise the importance of uncovering and addressing the effects of the pandemic on women and their families, and the potential for adverse physical, psychological, and psychosocial effects.

Women and maternity care providers recounted some positive experiences that arose from the alterations to care which warrant consideration for future maternity services. For example, as a result of visiting restrictions women recounted creating bonds with other women that might not have arisen otherwise, and having a calm space to establish breastfeeding. Maternity care providers recounted increased comradeship and bonding with their colleagues, which led to a more positive working environment. This will be important in the future as the pandemic endures whereby reliance and support from clinical colleagues will be more critical than ever. The structural and psychological challenges imposed by the pandemic on care providers has impacted on their reported ability to provide quality maternity care [86, 87]. Although few staff were reported as having considered resigning during the pandemic, the impact of the pandemic, and associated uncertainties, has taken its toll on providers physical and mental wellbeing [84]. To enable maternity care providers adequately support women in their care, and adequately support each other, appropriate training, resources (time and personnel) and equipment (e.g., adequate PPE) are required. Without such resources maternity care providers may continue to feel ill-equipped to provide the support that women need or desire or may lack confidence in being able to provide optimal maternity care.

### Strengths and limitations

The strength of this QES lies in the broad approach of including both women’s and maternity care providers’ views. This has enabled perspectives from both populations to be considered and compared in gaining insight and in understanding maternity care experiences in these unusual times. The rapidity with which studies on this topic were being published, however, could introduce a potential limitation for our findings. Although we updated our search prior to our synthesis, there is the likelihood that further studies will have since become available that meet the inclusion criteria for this QES. Reassuringly though, our updated search revealed only eight additional codes in total, from those already developed. In this sense, further studies are unlikely to significantly alter the findings of the QES. We accept however, that how maternity care has been experienced as the pandemic continued may have temporally changed, especially in the wake of the vaccination programme which may have helped reduce fears, lessened restrictions or impacted on how care was structured or provided. To assess for temporal trends, an update or, ideally, a second review from the time of our last search would be beneficial. A follow-on review would allow findings based on the initial year of the pandemic and later years to be compared or contrasted. We also acknowledge that maternity care experienced by women can be influenced by their partner’s feelings and experiences or by those of their wider family or circle of friends. To advance the evidence overall on the experience of maternity care, we plan to conduct a QES on partners’ experiences of maternity care during COVID-19 and explore further complementarity. A further limitation to our QES was the inclusion of studies in the English language only. To assess the potential for language bias, however, we unrestricted our search based on language. Seven records were retrieved that potentially might have meet the review’s inclusion criteria but were excluded as they were non-English language publications (see https://osf.io/bzt38/ for the list of excluded studies with reasons). Given the extent of data contributing to the QES, however, we feel reassured that the potential for language bias based on the exclusion of these records is minimal.

## Conclusion

Although some positives were identified, overall, this QES reveals that maternity care during COVID-19 was negatively experienced by both women and maternity care providers. Strong emotive states, many of which were prolonged, especially for maternity care providers, have the potential to impact on the future health and wellbeing of women, their families and that of maternity care providers. Resource and care planning (e.g., employee organised and funded mental health, resilience or debriefing workshops for staff, longer term postnatal follow-up and care for women) to mitigate such risks are required. To add further understandings of the experience of maternity care during COVID-19 overall, and to explore temporal trends and complementarity, additional QES updating the current QES and a QES to explore the views and experiences of partners and support persons are recommended; the latter of which is currently being planned.

## Supplementary Information

**Additional file 1:** **Supplementary File 1.** Minimum criteria for quality assessment (adaptedfrom Thomas [21] *et al.* 2003)

**Additional file 2: Supplementary File 2. **Methodological characteristics of included studies  

**Additional file 3:** Evidence Profile - GRADE CERQual

## Data Availability

All data are available in the manuscript or in the Additional files. The corresponding author can be contacted for additional information if required.

## Abbreviations

ENTREQ: Enhancing transparency in reporting the synthesis of qualitative research
EPPI: Evidence for Public and Practice Information
GRADE-CERQual: Grading of Recommendations Assessment, Development and Evaluation-Confidence in the Evidence from Reviews of Qualitative research
NICU: Neonatal intensive care unit
PPE: Personal protective equipment
QES: Qualitative evidence synthesis
UK: United Kingdom
USA: United States of America
