# Supplementary material for: Maternity care during COVID-19: a qualitative evidence synthesis of women’s and maternity care providers’ views and experiences
Source: BMC Pregnancy Childbirth. 2022 May 26;22:438. doi: 10.1186/s12884-022-04724-w (PMC9132752; doi:10.1186/s12884-022-04724-w)
Supplement: Supplementary file 1 — Additional file 1: Supplementary File 1. Minimum criteria for quality assessment (adaptedfrom Thomas [21] et al. 2003) [file 12884_2022_4724_MOESM1_ESM.docx]

**Additional File 1: Minimum criteria for quality assessment (adapted from Thomas *et al.* 2003)**

| ***Quality Criteria*** | | ***Comments (if useful)*** |
| --- | --- | --- |
| ***Quality of study reporting*** | A - Aims and objectives clearly reported | Y = Clear statement of aim is provided |
|  | B - Adequately described the context of the research | Y = Adequate background to the study provided |
|  | C - Adequately described the sample & sampling methods | Y = At a minimum the report must include demographics on numbers of primiparous/multiparous women, age of participants (either range or mean), and gestational age (range/mean). Similarly, for maternity care providers details on professional group (i.e., midwives, doctors, etc.,), age range/mean and years of practice (range/mean)  P = Some, but not all of the above  N = No information other than numbers taking part  Sampling methods  Y = Broadly described but explicitly stated (e.g., purposive, convenience, snowball, etc).  P = Some detail, but description of specific broad approach not provided  N = No information at all provided as to sampling approach |
|  | D - Adequately described the data collection methods | Y = explicitly described |
|  | E - Adequately described the data analysis methods | Y = explicitly described |
| ***There was good or some attempt to establish the*** | F - Reliability of the data collection tools | Qualitative study: Y for use of interview guide and what informed this  If a Survey was used, completely opened-ended response option(s) or PPI involvement = Y  Directed free-response option(s) maybe P or N depending on how broad the directed question(s) is and how the questions were developed (PPI or research team involvement, etc.,) |
|  | G - Validity of the data collection tools | As above |
|  | H - Reliability of the data analysis | Refers to analysis of qualitative data only  Y = A valid (e.g., thematic analysis, etc.,) approach described  P = Some details and appears like a valid approach but not explicit  N = Appears formal/structured but explicit details not provided |
|  | I - Validity of the data analysis | As above |
| ***Quality of the methods*** | J - Used the appropriate data collection methods to allow for expression of views | Y = For qualitative study (unless questions are very structured might be P or N)  If a survey – default to N with judgement on P or Y dependent on the broadness of open-ended response option(s) |
|  | K - Used the appropriate methods for ensuring the analysis was grounded in the views | Linked to H and I above – if valid and reliable data analysis this will be Y |
|  | L - Actively involved the participants in the design and conduct of the study | Y = Clear statement of PPI involvement from the outset (informing design/protocol development, etc.) |

*Y: Yes, N: No, P: Partially
