# Supplementary material for: Maternity care during COVID-19: a qualitative evidence synthesis of women’s and maternity care providers’ views and experiences
Source: BMC Pregnancy Childbirth. 2022 May 26;22:438. doi: 10.1186/s12884-022-04724-w (PMC9132752; doi:10.1186/s12884-022-04724-w)
Supplement: Supplementary file 2 — Additional file 2: Supplementary File 2. Methodological characteristics of included studies [file 12884_2022_4724_MOESM2_ESM.docx]

**Supplementary File 2: Methodological characteristics of included studies**

| **Reference** | **Study design** | **Study dates** | **Sampling method** | **Data collection method** | **Data analysis method** | **Funding** |
| --- | --- | --- | --- | --- | --- | --- |
| **Studies contributing data on women’s views and experiences** | | | | | | |
| Anonymous 2020 | Case report (personal story) | Jun 2020 | Not explicit | Narrative report of lived experience | Personal narrative | Not reported |
| Atmuri 2021 | Qualitative descriptive | 01 Jun-19 Jun 2020 | Convenience | Semi-structured interviews via telephone or video conferencing | Thematic analysis | Not reported |
| Aydin 2021 | Qualitative descriptive | Dec 2020 | Purposive | Semi-structured interviews | Thematic analysis | Not reported |
| Barbosa-Leiker 2021 | Cross-sectional survey | 28 Apr-30 Jun 2020 | Not explicit | Online questionnaire with open-text questions | Qualitative content analysis | Washington State University and the Drug and Alcohol Research Program |
| Bremen 2020 | Survey | 20 May-03 Jun 2020 | Convenience | Questionnaire with open-text response options | Content analysis | University of Maryland, Baltimore, Institute for Clinical and Translational Research |
| Castelino 2020 | Case report (personal story) | Not stated | Not explicit | Narrative report of lived experience | Personal narrative | Not reported |
| Cooper 2021 | Survey (preliminary findings) | 06 Apr-01 May 2020 | Purposive | Online national survey with open-text response options | Thematic analysis | Not reported |
| Cullen 2021 | Survey | Jul 2020 | Convenience | On-site paper questionnaire with open-text response options | Thematic analysis | Not reported |
| Einion-Waller 2021 | Survey | Mar-Aug 2020 | Not explicit | Questionnaire with open-text questions | Thematic analysis | Not reported |
| Farewell 2020 | Mixed methods (qualitative followed by quantitative) | Mar-Apr 2020 | Purposive | Telephone interviews | Constant comparative method | The author(s) received no financial support for the research, authorship, and/or publication of their study |
| Farrell 2021 | Qualitative descriptive | May-Jul 2020 | Convenience | Telephone interviews (structured) | Inductive process consistent with GT | Not reported |
| Fumagalli 2021 | Qualitative study using interpretive phenomenology | Mar-Jun 2020 | Purposive | Semi-structured interviews conducted by telephone/video-call or face-to face depending on the woman’s preference | Thematic analysis | Self-funded |
| Gomez-Roas 2021 | Qualitative descriptive | Not stated | Not explicit | Semi-structured telephone interviews | Constant comparative method | Not reported |
| Javaid 2021 | Cross-sectional survey | 03 Apr-24 Apr 2020 | Convenience and snowball | Online questionnaire with open-text response options | Attride-Sterling Framework | *‘This research did not receive any specific grant from funding agencies in the public, commercial, or not-for-profit sectors’* |
| Karavadra 2020 | National survey | 01-09 May 2020 | Not explicit | Online questionnaire (national) | Thematic analysis | *‘No funding was required’* |
| Kumari 2021 | Qualitative descriptive | Sept 2020 | Purposive with maximum variation | Focus group discussion (3 x 5 in each, one online, two on the antenatal ward) and 10 individual interviews | Qualitative content analysis | Supported by AIIMS New Delhi under Fast track intramural research grant theme: research on SARS-COV-2 AND COVID-19 |
| Meaney 2021 | Cross-sectional survey | 16 Jun-17 Jul 2020 | Not explicit | Questionnaire with four open-ended questions | Qualitative content analysis | Supported by ESI and HRB-TMRN COVID-19 Emergency Evidence Response Service Summer Scholarship |
| Mortazavi 2021 | Descriptive phenomenological approach | 10-20 May 2020 | Purposive (variation sampling) | In-depth semi-structured interviews via the WhatsApp social network | Colaizzi’s seven-step content analysis method | Funded by the university which approved the proposal |
| Panda 2021 | Qualitative descriptive | 15 Sept-23 Oct 2020 | Purposive | Semi-structured interviews via telephone | Thematic analysis | Office of the Dean of Research, Trinity College Dublin, COVID Response Fund |
| Perez 2021 | Cross-sectional survey | 24 Apr-13 May 2020 | Not explicit | Online survey with open-text response options | Thematic analysis | This research received no specific grant from any funding agency, commercial or not-for-profit sectors |
| Rhodes 2020 | Mixed methods (web-based survey followed by interviews) | 29 Apr-18 Jun (interviews) | Initially random but later based on age, postcode, and ESL to ensure a representative sample | Semi-structured interviews via telephone | Framework and thematic analysis | Supported by the Economic and Social Research Fund and the UK charity Best Beginnings |
| Sahin 2021 | Qualitative descriptive study | Not stated | Snowball | Semi-structured questionnaire with open-ended questions | Qualitative content analysis | Not reported |
| Snyder 2021 | A cross-sectional phenomenological approach | Mar-Jun 2020 | Not explicit | Semi-structured interviews | Immersion and crystallization | Not reported |
| Spatz 2021 | Case series (of lived experiences) | Not stated | Convenience | Virtual interviews | Thematic analysis | Not reported |
| Sweet 2021 | Cross-sectional survey and qualitative interviews | Mar-Jun 2020 | Maximum variation | Semi-structured interviews via Zoom or telephone | Thematic analysis | Institute for Health Transformation, Faculty of Health, Deakin University |
| Upendra 2020 | Phenomenology | Not stated | Purposive | In-depth interview supplemented with observational notes | Colaizzi’s seven steps of phenomenological data analysis | Self-funded |
| Wallace 2020 | Case report (personal story) | Not stated | Not explicit | Personal narrative | Personal narrative | Not reported |
| **Studies contributing data on maternity care professional’s views and experiences** | | | | | | |
| Bradfield 2021 | Cross-sectional survey and qualitative interviews | 13 May-24 Jun 2020 | Convenience with maximum variation | Semi-structured interviews | Thematic analysis | Not reported |
| Burns 2020 | Case report (of lived experience) | May 2020 | Not described | Personal narrative of lived experience | Personal narrative | Not reported |
| Claudio 2020 | Qualitative commentary | Not stated | Convenience | Personal narratives of experiences | Personal narratives organized by themes | Not reported |
| Danvers 2020 | Qualitative commentary | Mar 2020 | Not described | Personal narratives of lived experience | Personal narratives | Not reported |
| Dulfe 2021 | Descriptive and exploratory qualitative | Feb-Apr 2020 | Convenience | Semi-structured interviews | Content analysis | Ministry of Health, Brazil |
| Elsayed 2021 | Cross-sectional survey | 01 Apr-15 May 2020 | Not described | Questionnaire with open-text response options | Not stated | Not reported |
| Galle 2021 | Cross-sectional survey | Jul-Sept 2020 | Non-random and snowball | Online questionnaire with open-text response options | Thematic analysis | Institute of Tropical Medicine’s COVID-19 Pump Priming fund supported by the Flemish Government, Science and Innovation |
| Gonzalez-Timoneda 2020 | Qualitative study with a phenomenological approach | May-Jun 2020 | Purposive | Individual open-ended interviews | Giorgi’s four-step phenomenological approach | *‘Not applicable’* |
| Homer 2021 | Cross-sectional survey | Apr 2020 | Convenience | Online questionnaire with open-text response options | Content analysis | Funded internally through the Burnet Institute |
| Kang 2021 | Descriptive qualitative | Aug-Nov 2020 | Purposive | Focus group interviews (n=8) and individual in-depth interviews (n=4) | Content analysis | *‘The authors received no specific funding for this work’* |
| Madden 2020 | Mixed methods (survey followed by interviews) | 09 Mar-12 Apr 2020 | Not described | Semi-structured interviews (via Zoom with the Voice Memos application) | Framework approach using thematic analysis | *‘None’* |
| Oparah 2021 | Qualitative study | Jul 2020 | Purposive | Sharing circles held virtually over Zoom to collect narratives | Thematic analysis | Not reported |
| Peahl 2021 | Survey | 28 Mar-07 Apr 2020 | Convenience | Interview delivered questionnaires with open-text response options | Thematic analysis | Not reported |
| Reyes 2021 | Qualitative study | Apr-Aug 2020 | Not described | Individual interviews via Zoom or telephone | Not explicit (although refers to *‘themes’*) | Not funded |
| Schindler-Ruwisch 2021 | Cross-sectional survey | Jun 2020 | Purposive | Online survey with open-text response options | Thematic analysis | Internal research grant from Fairfield University |
| Semaan 2020 | Cross-sectional survey | 24 Mar-10 Apr 2020 | Non-random and snowball | Online survey in 12 languages with open-text response options | Thematic analysis | Institute of Tropical Medicine’s COVID-19 Pump Priming fund supported by the Flemish Government, Science & Innovation |
| Szabo 2021 | Cross-sectional survey and qualitative interviews | May-Jun 2020 | Convenience | Semi-structured interviews | Thematic analysis | Internal funding from the Burnet Institute funded transcriptions |
| **Studies contributing data on both women’s and professionals’ views and experiences** | | | | | | |
| Altman 2021 | Qualitative descriptive | Apr-Aug 2020 | Targeted and snowball | Unstructured virtual interviews via Zoom | Thematic analysis | University of Washington School of Nursing, Seattle |
| Bender 2020 | Retrospective cohort study | 13-26 Apr 2020 | Convenience | Semi-structured interviews (women) and online survey (HCPs) | Thematic analysis | Not reported |
| Bengalia 2021 | A short-term ethnographic research plan | 04 Jul-04 Aug 2020 | Purposive (women) and convenience (MCPs) | Two online questionnaires, in-depth conversations, informal exchanges with local birth providers, and participation in relevant webinars | Not stated | None reported |
| Hailemariam 2021 | Exploratory descriptive design | 25 Sept-25 Nov 2020 | Convenience (women) and Purposive (MCPs) | Focus group discussion (women) and individual interviews (HCPs) | Thematic analysis | No financial support was received for the study or publication |
| Ombere 2021 | Rapid qualitative study | 13 Jun-24 Jul 2020 | Purposive | Interviews via telephone | Not explicit | Netherlands organization for scientific research-WOTRO science for Global Development Grant and Swiss Tropical and Public Health Institute Switzerland |
| Peahl 2021 | Survey | 16 Dec 2019-28 Jun 2020 | Convenience | Questionnaire with open-text response options | Thematic analysis | National Institute on Drug Abuse, the Agency for Healthcare Research and Quality, AAOG Foundation, the Laura and John Arnold Foundation, National Institute for Reproductive Health, the Blue Cross Blue Shield of Michigan Foundation, and the NIH |
