# Supplementary material for: Maternity care during COVID-19: a qualitative evidence synthesis of women’s and maternity care providers’ views and experiences
Source: BMC Pregnancy Childbirth. 2022 May 26;22:438. doi: 10.1186/s12884-022-04724-w (PMC9132752; doi:10.1186/s12884-022-04724-w)
Supplement: Supplementary file 3 — Additional file 3: Evidence Profile - GRADE CERQual [file 12884_2022_4724_MOESM3_ESM.docx]

**Additional File 3: Evidence Profile - GRADE CERQual**

| **Finding** | **Contributing records** | **Methodological limitations:** *quality of the studies which are contributing to finding* | **Coherence:** *extent of support for review finding from the underlying data* | **Adequacy:** *richness of data; depth and quantity contributing to the finding; includes design* | **Relevance:** *inclusion criteria for studies should closely mirror the review question; study aim, population and focused aspect of COVID-19 that is explored considered* | **Overall Confidence** |
| --- | --- | --- | --- | --- | --- | --- |
| **Analytical theme 1: Altered Maternity Care (women)** | | | | | | |
| Alterations to maternity care, overall, were unsettling for women, causing increased stress, anxiety, worry, uncertainty, or dissatisfaction | 38, 40, 41, 46, 49, 50, 52, 54-56, 57 | **Minor concerns:** most contributing studies were of high quality, though one study (41) met only five quality criteria | **No or very minor concerns;** some studies do report positive views on alterations to care (e.g., 40) but overall, this finding is consistent across the studies | **Minor concerns:** contributing studies seem to provide relatively equal amounts and depth of data. Four studies were survey designs; and data was also generated in mixed-methods and case-series/studies. Two used phenomenology while one conducted interview in addition to a survey | **Minor concerns:** majority of studies are well aligned with the review question and aim, though Einion Waller focused on hypnobirthing, Meaney had a specific focus on social-support, care, and stress-reduction, and Snyder focused on social support; populations included well aligned. | **High** |
| Uncertainty and inconsistencies surrounding maternity care were a considerable source of stress, anxiety, frustration, and dissatisfaction for women | 2, 34, 37, 39, 42, 44, 49, 56, 79 | **Minor concerns:** most contributing studies were of high quality | **Minor concerns:** the data were varied for this finding.  Wallace and Einion Waller refer to positive aspects, though overall findings are fairly consistent | **Minor concerns:** contributing studies seem to provide relatively equal amounts and depth of data; varying levels of data, with four studies reporting on survey data (Cooper, Meaney, Bremen, Peahl); and one reporting on mixed-methods date (Farewell) | **Minor concerns:** the study aims, and populations are generally well aligned, however: Farewell focuses on mental health and wellbeing and sources of resilience; Fumagalli focuses on covid-19 positive mothers; Peahl has a specific focus on a covid-19 care model. | **High** |
| Cancelled or postponed maternity care appointments were commonly experienced leaving women feeling confused, worried, fearful, and abandoned | 2, 34, 35, 39, 43, 45-47, 49-52 | **Minor concerns**: most contributing studies were of high quality, though one study (Gomez-Roas) met only five quality criteria | **No or very minor concerns**; this finding is consistent across the studies | **Moderate concerns:** data seems to come largely from subgroup of studies; varying designs used, includes five surveys | **Minor concerns:** the study aims, and populations are generally well aligned, although Farrell focuses on prenatal genetic screens and diagnostic tests; Gomez-Roas focuses on challenges within healthcare interactions | **Moderate** |
| Telehealth was noted to confer some benefits; overall, however, telehealth was problematic for women and was favoured less than in-person care | 2, 33, 34, 39, 42, 45-48, 52, 54, 55, 74, 75, 79 | **Minor concerns**: most contributing studies were of high quality, though one study (Gomez-Roas) met only five quality criteria | **No or very minor concerns**; even though there is a difference between the benefits and the problematic aspects, there is relative coherence across the studies | **Minor concerns:** data depth and quantity contributing to finding seems relatively equal across studies. Varying study designs, though some more in-depth qualitative examinations; also includes surveys, cohorts. | **Minor concerns;** the study aims, and populations are generally well aligned, although Peahl has a specific focus on a covid-19 care model; Farewell focuses on mental health and wellbeing and sources of resilience; Snyder focuses on social support; Gomez Roas focuses on challenges within healthcare interactions; and Bender focused on asymptomatic women tested for SARS-CoV-2 | **Moderate** |
| **Analytical theme 2: COVID related restrictions** | | | | | | |
| Restrictions on partner attendance throughout the maternity care continuum evoked a wide array of emotions for women including intense feelings of being alone, isolated, and lonely | 2, 38-39, 42-44, 46, 47, 49, 52, 55, 56, 58, 76 | **Minor concerns**: most contributing studies were of high quality, though one study (Benaglia) met only four quality criteria | **No or very minor concerns**; this finding is consistent across the studies, although there was variation in the level of restrictions on partners, and the emotions experienced vary across the studies | **Moderate concerns:** Varying study designs (with use of surveys), though data depth and quantity contributing to finding seems relatively equal across studies. | **Minor concerns:** generally, the studies aims, and populations are well aligned, although: Fumagalli focuses on covid-19 positive mothers; Farewell focuses on mental health and wellbeing and sources of resilience; Farrell focuses on prenatal genetic screens and diagnostic tests; and Cooper focuses on informing government. | **Moderate** |
| Isolation and separation from friends and the wider family affected women in various ways (disappointment, loneliness, fear, anxiety, overwhelmed), although the wider visiting restrictions in hospital beyond partner visiting, was a positive experience for some women | 2, 34, 36-38, 40-44, 47, 49, 50, 51, 55, 56 | **Minor concerns**: most contributing studies were of high quality, though one study (Einion-Waller) met only five quality criteria | **Minor concerns:** data is fairly consistent though there is inconsistency in the studies reporting positive versus negative aspects (e.g. Cullen & Panda focus on positive aspects only) | **Moderate concerns:** Varying study designs, though data depth and quantity contributing to finding seems relatively equal across studies. Also Panda and Cullen could be considered to contribute a bit more here than other studies. | **Minor concerns:** the study aims, and populations are well aligned, although: Farewell focuses on mental health and wellbeing and sources of resilience; Farrell focuses on prenatal genetic screens and diagnostic tests; Cullen focuses on visiting restrictions | **Moderate** |
| **Analytical theme 3: Infection prevention and risk** | | | | | | |
| Fear of contracting COVID-19 was prominent for women, with many fearful, worried, and wary of visiting the maternity care facility for fear of contracting the virus | 34-36, 39, 41, 43, 44, 47, 48, 50, 52, 55, 57, 75, 77 | **Minor concerns**: most contributing studies were of high quality, though one study (Einion-Waller) met only five quality criteria | **No or very minor concerns**; this finding is consistent across the studies | **Moderate concerns:** Varying study designs (with use of surveys), though data depth and quantity contributing to finding seems relatively equal across studies. | **Minor concerns:** the study aims, and populations are generally well aligned. Although Farrell focuses on prenatal genetic screens and diagnostic tests; Barbosa focuses on stressors, coping behaviors, and resources; Fumagalli focuses on covid-19 positive mothers; Einion-Waller has a specific focus on hypnobirthing online classes. | **Moderate** |
| The interplay between balancing fear of contracting COVID-19 and the risk of not attending for care was a source of emotional conflict | 39, 46, 77 | **No or very minor concerns**: most contributing studies were of high quality | **No or very minor concerns;** this finding is consistent across the studies | **Moderate concerns;** data from surveys x 2 and one focus group study. | **Minor concerns,** Majority well aligned, though Cooper focuses on informing government; Einion Waller focuses on hypnobirthing classes. | **High** |
| Women were complementary and appreciative of efforts in maternity care settings to minimise virus transmission and felt reassured by these | 2, 34, 40, 44-46, 52, 58, 75 | **Minor concerns**: most contributing studies were of high quality, though one study (Gomez-Roas) met only five quality criteria | **No or very minor concerns**; this finding is consistent across the studies | **Moderate concerns:** Varying study designs (with use of surveys), though data depth and quantity contributing to finding seems relatively equal across studies. | **Minor concerns,** Majority well aligned, though Bender focuses on asymptomatic obstetric patients tested for SARS-CoV-2; Cullen looked more specifically at visiting restrictions | **Moderate** |
| **Analytical theme 4: *“The lived reality” –* navigating support systems** | | | | | | |
| Information support was affected by a lack of consistent messaging, conflicting information or a lack of clear guidance surrounding the virus and how this affected women’s care, which left women feeling lost, confused, or helpless | 37, 39, 42, 46, 49,  51, 53, 55, 56 | **Minor concerns**: some of the studies only partially met some of the quality criteria but most contributing studies were of high quality. | **No or very minor concerns**; this finding is consistent across the studies. | **Moderate concerns:** five of the contributing studies are based on open-ended survey responses which may lack sufficient depth; the remaining studies are of different design but appear to provide depth of data. | **Minor concerns**: generally, the aims and populations align with the review question, although Farewell focuses on mental health and Perez focuses on mood and parenting confidence. | **Moderate** |
| Women viewed dedicated formal support from maternity care professionals as essential for their psychosocial wellbeing; however, these supports were largely diminished or lacking | 2, 34, 36, 37, 41, 42, 44, 45, 49, 51, 55, 56, 74 | **Minor concerns**: some of the studies only partially met some of the quality criteria but most were of high quality. | **No or very minor concerns**; some studies do report good support (Cullen, Spatz, Wallace), however overall, this finding appears to be consistent across the studies. | **No or very minor concerns**; four of the contributing studies are based on open-ended survey responses which may lack sufficient depth; the remaining studies are of different design but appear to provide depth of data. | **Minor concerns:** generally, the aims and populations align with the review question, although Barbosa focuses on stressors, coping behaviors, and resources; Fumagalli focuses on covid-19 positive mothers; Farewell focuses on mental health; Einion-Waller has a specific focus on hypnobirthing online classes; Gomez Roas focuses on challenges within healthcare interactions; and Perez focuses on mood and parenting confidence. | **High** |
| In navigating information support, many women resorted to alternative sources, mainly social media, television, and online sources, as well as friends, although women recognised that these alternative sources could be unreliable which caused stress and fear | 34, 38-40, 44, 47, 50, 52, 53, 55, 56 | **Minor concerns**: most contributing studies were of high quality. | **No or very minor concerns**; this finding is consistent across the studies. | **Minor concerns**: two of the contributing studies are based on open-ended survey responses which may lack sufficient depth; but the majority provide rich data. | **No or very minor concerns**; the aims and populations align with the review question, although Fumagalli focuses on covid-19 positive mothers. | **High** |
| Women self-implemented solutions as a means of coping, including adjusting their plans, exploring other options for care or self-advocating to achieve the maternity care they desired or needed | 2, 37, 39, 47, 56 | **Minor concerns**: most contributing studies were of high quality. | **Minor concerns:** this finding is consistent across the studies, although four studies report women accepting the situation in order to feel prepared | **Moderate concerns:** four of the contributing studies (over half) are based on open-ended survey responses which may lack sufficient depth. | **No or very minor concerns**; the aims and populations align with the review question. | **Moderate** |
| **Analytical theme 5: Interactions with maternity services** | | | | | | |
| Women recounted being unable to contact or experienced fewer interactions with their care providers which led women, in general, to view their care as inadequate, sub-par, disrespectful or of poorer quality | 2, 36, 37, 39, 41, 43-47, 49, 52, 53,  74, 75 | **Minor concerns:** five of the contributing studies failed to meet all of the quality criteria or only partially met many of the criteria | **No or very minor concerns**; this finding is consistent across the studies. | **Moderate concerns:** seven of the contributing studies (almost half) are based on open-ended survey responses which may lack sufficient depth; the remaining appear to be of adequate richness. | **Minor concerns**: The aims and populations generally align with the review question, although Barbosa focuses on stressors, coping behaviors, and resources; Einion-Waller has a specific focus on hypnobirthing online classes; Fumagalli focuses on covid-19 positive mothers; Gomez Roas focuses on challenges within healthcare interactions; Farrell focuses on prenatal genetic screens and diagnostic tests. | **Moderate** |
| Some women who were positive for COVID-19 experienced what they perceived as nonprofessional and inappropriate interactions | 39, 44, 46, 47, 52, 55, 56, 77 | **Minor concerns**: most contributing studies were of high quality. | **No or very minor concerns**; this finding is consistent across the studies. | **Minor concerns**: three of the contributing studies are based on open-ended survey responses which may lack sufficient depth; but the majority provide rich data. | **No or very minor concerns**; the aims and populations align with the review question, although Fumagalli focuses on covid-19 positive mothers. | **High** |
| Women experienced unmet expectations arising from interactions with their maternity care providers source which affected their ability to prepare properly for the arrival of their new baby | 39, 40-42, 44, 56 | **Minor concerns**: two of the studies only partially met the quality criteria but most were of high quality. | **No or very minor concerns**; this finding is consistent across the studies. | **Moderate concerns:** three of the contributing studies are based on open-ended survey responses which may lack sufficient depth. | **Minor concerns**: generally, the aims and populations align with the review question, although Farewell focuses on mental health; Einion-Waller focuses on those who accessed a hypnobirthing class; and Fumagalli focuses on covid-19 positive mothers. | **Moderate** |
| **Analytical theme 6: Altered Maternity Care (maternity care providers)** | | | | | | |
| A feeling of uncertainty was dominant across providers, largely influenced by the uncertainty surrounding care protocols and the speed at which these changed, although this uncertainty lessened over time as national guidelines became available and communication of care protocols improved | 17, 60-63, 65, 66, 68, 70, 72-74 | **Minor concerns:** four contributing studies failed to meet all quality criteria or only partially met many criteria, and one study only met one quality criterion. | **No or very minor concerns**; this finding is consistent across the studies. | **Minor concerns**: three of the contributing studies are based on open-ended survey responses which may lack sufficient depth; but the majority provide rich data. | **No or very minor concerns**; the aims and populations align with the review question, although Homer focuses private practising midwives. | **High** |
| The pandemic was considered to have exacerbated existing inequalities in maternity care | 61, 64,67-69, 71, 72, 74 | **Minor concerns:** three of the contributing studies failed to meet all of the quality criteria or only partially met many criteria, and one study only met one quality criterion. | **No or very minor concerns**; this finding is consistent across the studies. | **No or very minor concerns**; two of the contributing studies are based on open-ended survey responses which may lack sufficient depth; but the majority provide rich data. | **Minor concerns**: generally, the aims and populations align with the review question, although Madden focuses on telehealth and Schindler-Ruwisch focuses on breastfeeding support services. | **High** |
| The lack of access to adequate resources and training on safe practices resulted in providers limiting their interactions with women as they feared being infected and/or acting as a vector of infection | 17, 63, 65, 66, 70, 74, 77 | **No or very minor concerns**; one study only met one quality criterion, but the majority were of high quality. | **No or very minor concerns**; this finding is consistent across the studies. | **Minor concerns**: two of the contributing studies are based on open-ended survey responses which may lack sufficient depth; but the majority provide rich data. | **No or very minor concerns**; the aims and populations align with the review question, although Homer focuses private practising midwives. | **High** |
| A move to telehealth was viewed positively by some as it enabled continuation of care in a safe environment, although it was not without its limitations | 64, 67-69, 71 | **Minor concerns:** two of the contributing studies only partially met many of the criteria, and one of the studies (Reyes, 2021) only met one quality criterion. | **Minor concerns**: Some distinctions in views based on positive and limiting aspects. | **Moderate concerns:** two of the five contributing studies are based on open-ended survey responses which may lack sufficient depth; and a lot of data seems to come from two studies | **Minor concerns;** the aims and populations generally align with the review question, although Madden focuses on telehealth, Schindler-Ruwisch focuses on breastfeeding support, Oparah focused on black birth workers. | **Moderate** |
| **Analytical theme 7: Professional and Personal Impact** | | | | | | |
| The pandemic had resulted in an increased workload for maternity care providers, due to staff shortages, additional tasks, and more frequent and longer appointments | 17, 59, 62, 64-66, 68, 69, 73 | **Minor concerns**: most contributing studies were of high quality, though one study (Reyes, 2021) only met one quality criterion. | **Moderate concerns;** Some differences depending on role | **Minor concerns:** data depth and quantity contributing to finding seems relatively equal across studies. Majority of data derived from qualitative studies, with one mixed-methods study and two surveys | **Minor concerns:** Oparah focused on black birth workers, Homer focused on private practicing midwives, Galle had a telemedicine focus, Szabo focused specifically on doctors. | **Moderate** |
| Relationships with colleagues improved as maternity care providers supported each other through the uncertainty, although the pandemic also deepened divisions due to perceived staff hierarchies and disconnect between management and providers involved in direct care | 15, 59-63, 65, 66, 69, 70, 72, 74, 76, 77 | **Moderate concerns:** two of the contributing studies only met one criterion, one only met four criteria, and all only partially. | **Minor concerns;** some differing data in relation to different healthcare settings, and also different beliefs or perceptions. | **Minor concerns**: two of the contributing studies are based on open-ended survey responses which may lack sufficient depth; but the majority provide rich data. | **No or very minor concerns**; the aims and populations align with the review question. | **Moderate** |
| Maternity care providers isolated themselves or restricted their interactions with family due to a fear of transmitting the virus to others, which carried a significant emotional burden | 17, 59, 61, 65, 66, 68, 69, 73, 77, 78 | **Minor concerns:** two of the contributing studies only met one of the quality criteria, and one study only met two criteria, both partially. | **No or very minor concerns**; this finding is consistent across the studies. | **Minor concerns**: two of the contributing studies are based on open-ended survey responses which may lack sufficient depth; but the majority provide rich data. | **Minor concerns:** Majority seem well aligned, though Oparah focused on black birth workers, Homer focused on private practicing midwives, Szabo focused on doctors only, | **High** |
| The pandemic had a negative financial impact for some providers due to reduced service demand and inadequate reimbursement for alternative services, such as telehealth | 64, 68, 73 | **No or very minor concerns**; most contributing studies were of high quality. | **No or very minor concerns**; this finding is consistent across the studies. | **Moderate concerns**: based on mixed designs which potentially contribute imbalanced data depth and quality, e.g., Szabo is mixed-methods, Oparah is qualitative, Galle is a survey. | **Moderate concerns:** Oparah focused on black birth workers only, Szabo focused on doctors only, Galle focused on telemedicine | **Moderate** |
| **Analytical theme 8: Broader structural impact** | | | | | | |
| Restrictions were considered by some to have a negative impact on future health outcomes for parents and babies, while others worried that certain restrictions would be retained, and these would negatively influence future maternity care | 59, 69, 72, 76 | **Moderate concerns**: One study met only one quality criteria; one study met only four criteria and remaining evidence from two other studies | **No or very minor concerns**; this finding is consistent across the studies. | **Minor concerns**: one of the four contributing studies is based on open-ended survey responses which may lack sufficient depth; but the majority provide rich data. | **No or very minor concerns**; the aims and populations align with the review question. | **High** |
| The pandemic was viewed as an opportunity to improve maternity care, including addressing inequalities, and implementing changes that supported parents and their babies | 15, 59, 61, 69, 73, 76, 78 | **Moderate concerns:** two of the contributing studies only met one criterion, and one study only met two criteria, both partially. | **No or very minor concerns**; this finding is consistent across the studies. | **No or very minor concerns**; the majority of contributing studies provide rich data. | **Minor concerns**: the aims and populations align with the review question, although Szabo focused on doctors only. | **High** |
| The pandemic prompted some maternity care providers to take a different perspective of their role and considered it an opportunity for professional growth | 59, 61, 66, 68, 69, 76 | **Minor concerns:** one of the studies only met one of the quality criteria, and one study only met four criteria, and all only partially. | **No or very minor concerns**; this finding is consistent across the studies. | **Minor concerns:** data depth and quantity contributing to finding seems relatively equal across studies, with majority of data from qualitative studies | **Minor concerns**: the aims and populations align with the review question, although Oparah focused on black birth workers only, | **Moderate** |
